# Interaction between endogenous microRNAs and virus-derived small RNAs controls viral replication in insect vectors

**DOI:** 10.1371/journal.ppat.1010709

**Published:** 2022-07-07

**Authors:** Wan Zhao, Qiong Li, Mengqi Sun, Yan Xiao, Feng Cui

**Affiliations:** 1 State Key Laboratory of Integrated Management of Pest Insects and Rodents, Institute of Zoology, Chinese Academy of Sciences, Beijing, China; 2 CAS Centre for Excellence in Biotic Interactions, University of Chinese Academy of Sciences, Beijing, China; North Carolina State University, UNITED STATES

## Abstract

MicroRNAs (miRNAs) play an important role in resisting virus infection in insects. Viruses are recognized by insect RNA interference systems, which generate virus-derived small RNAs (vsRNAs). To date, it is unclear whether viruses employ vsRNAs to regulate the expression of endogenous miRNAs. We previously found that miR-263a facilitated the proliferation of rice stripe virus (RSV) in the insect vector small brown planthopper. However, miR-263a was significantly downregulated by RSV. Here, we deciphered the regulatory mechanisms of RSV on miR-263a expression. The promoter region of miR-263a was characterized, and the transcription factor YY1 was found to negatively regulate the transcription of miR-263a. The nucleocapsid protein of RSV promoted the inhibitory effect of YY1 on miR-263a transcription by reducing the binding ability of RNA polymerase II to the promoter of miR-263a. Moreover, an RSV-derived small RNA, vsR-3397, downregulated miR-263a transcription by directly targeting the promoter region with partial sequence complementarity. The reduction in miR-263a suppressed RSV replication and was beneficial for maintaining a tolerable accumulation level of RSV in insect vectors. This dual regulation mechanism reflects an ingenious adaptation strategy of viruses to their insect vectors.

## Author summary

RNA interference (RNAi) plays a key role in intrinsic cellular immunity to viruses in invertebrates. In addition to endogenous small RNAs, virus-derived small RNAs (vsRNAs) are synthesized in host cells. However, whether communication occurs between the two types of small RNAs remains elusive. Here, we report such a case in the system of rice stripe virus (RSV) and its insect vector small brown planthopper. The expression of insect endogenous microRNA miR-263a is inhibited not only by viral proteins but also by a vsRNA that affects the promoter function of miR-263a. This inhibitory effect is beneficial for maintaining a tolerable accumulation level of RSV in insect vectors, thus reflecting the ingenious adaptation strategy of viruses to their insect vectors.

## Introduction

In invertebrates and plant cells, RNA interference (RNAi) is mostly utilized for cell-intrinsic immunity to viruses [1, 2]. MicroRNAs (miRNAs) and small interfering RNAs (siRNAs) play predominant roles in resisting virus infection in insects [3]. These small RNAs function within the RNA-induced silencing complex (RISC), which contains different Argonaute (Ago) proteins, and usually guide the degradation of target RNAs or suppress the translation of proteins [4]. Insect endogenous miRNAs affect viral replication or assembly by regulating insect gene expression [5, 6] or directly targeting the untranslated terminal regions (UTRs) of viral genomic RNAs [7, 8]. Conversely, viruses can interfere with distinct steps of the RNAi pathway and influence the expression of host miRNAs [9, 10]. However, the regulatory mechanisms of viruses on the expression of host miRNAs remain elusive.

RNAi also plays an indispensable role in the sustainable infection of viruses in their insect vectors. Vector-transmitted plant viruses or arboviruses of human or animal viruses have evolved to maintain a tolerable accumulation level in their insect vectors to avoid high fitness costs, such as being pathogenic to vectors [11–13]. In addition to affecting the profile of endogenous miRNAs, viruses are recognized and utilized by vector RNAi systems to generate virus-derived small RNAs (vsRNAs) [14]. Although vsRNAs are generally thought to inhibit viral replication via complementarity to viral sequences, reports have indicated that vsRNAs are beneficial to virus infection by regulating the immune gene expression of hosts [14–16]. It is unclear whether crosstalk occurs between vsRNAs and endogenous miRNAs, such as the direct regulation of endogenous miRNA expression by vsRNAs.

Rice stripe virus (RSV) is a single-stranded RNA virus of the genus *Tenuivirus*. This virus is efficiently transmitted by the small brown planthopper *Laodelphax striatellus* in a persistent-propagative mode and has caused great economic damage to rice production in many East Asian countries [17]. The genome of RSV contains four RNA segments and encodes an RNA-dependent RNA polymerase (RdRp), a nucleocapsid protein (NP), and five nonstructural proteins (NS2, NSvc2, NS3, SP, and NSvc4) [18]. For the coordination between RSV and insect vector immune systems, in addition to inducing apoptosis reactions through binding the transcription factor YY1 in the nuclei of midgut cells and suppressing proteolytic activation of prophenoloxidase in the hemolymph [19], RSV generates a great number of vsRNAs and directly interacts with RNAi systems [14, 20]. Furthermore, we found that one endogenous miRNA, miR-263a, binds the 3’ terminal extension sequence of one genomic RNA of RSV to promote virus replication by eliminating the adverse effect of 3’ extensions on viral promoter activity in small brown planthoppers [21]. Surprisingly, RSV infection significantly downregulates the expression of miR-263a in midgut cells based on unknown mechanisms [21]. In the present study, we deciphered the regulatory mechanisms of RSV on the expression of miR-263a and showed that viral proteins and vsRNAs jointly downregulated the expression of miR-263a to control RSV replication in insect vectors.

## Results

### Identification of the miR-263a promoter region

**Fig 1:**
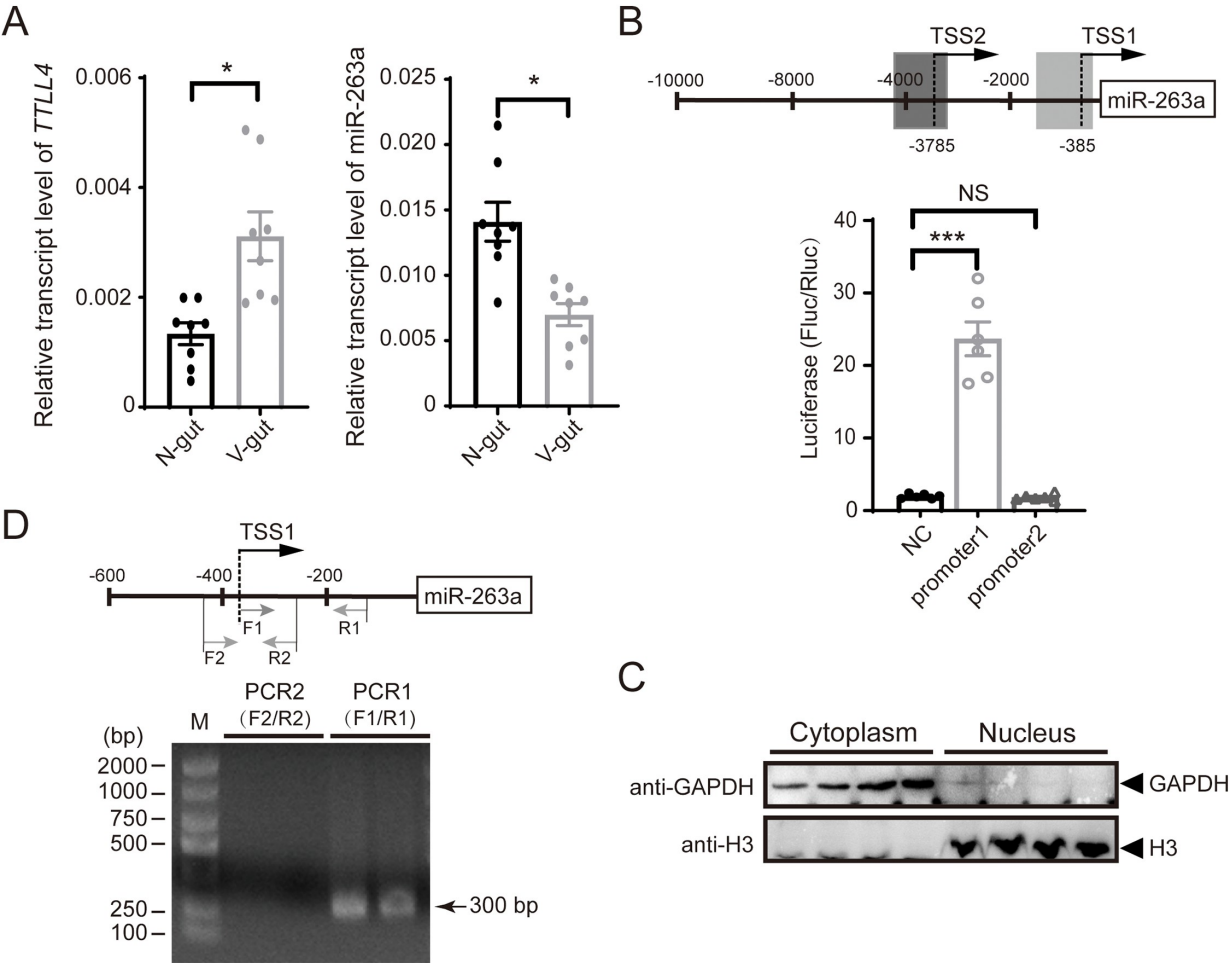
Determination of miR-263a promoter region. (A) Relative transcript levels of *tubulin polyglutamylase* (*TTLL4*) and miR-263a in the gut of nonviruliferous (N) and viruliferous planthoppers (V) (n = 8). The planthopper *EF2* gene and U6 snRNA were used as endogenous controls. (B) Promoter activity of recombinant pGL4.10 plasmids containing one of two putative promoter regions of miR-263a in human 293T cells in the dual luciferase reporter assay (n = 6). For (A) and (B), comparison was analyzed by Student’s t test. NS, no significant difference. *, P<0.05. ***, P<0.001. The relative activity of firefly luciferase (Fluc) to *Renilla* luciferase (Rluc) is presented. Empty pGL4.10 was used as the negative control (NC). The schematic diagram shows two candidate transcription start sites (TSSs), from which putative promoter regions were cloned. The 5’ terminal nucleotide of mature miR-263a is designated as position +1. NS, no significant difference. Data in (A) and (B) show the mean values and standard errors and were compared by Student’s t test. (C) Western blot analysis of the nuclear and cytoplasmic extracts from viruliferous planthoppers. (D) Reverse transcription-PCR of the putative transcripts starting exactly from TSS1 or from 96 bp upstream of TSS1 with the cDNA template of the nuclear extracts. Two replicates are shown. Schematic diagram shows the positions of PCR primers. M, marker.

The promoter region of miR-263a was identified to study its regulation. According to the genome information of the small brown planthopper [22], miR-263a is located at the 18^th^ intron of the *tubulin polyglutamylase* (*TTLL4*) gene (registration number MW353860). The transcript level of *TTLL4* was upregulated in the guts of viruliferous planthoppers compared to that of nonviruliferous planthoppers, which was opposite to the expression pattern of miR-263a (Fig 1A). This finding demonstrates that miR-263a has an independent promoter that is not shared with its host gene *TTLL4*.

Then, a 10 kb sequence upstream of miR-263a was obtained from *L*. *striatellus* genome (GenBank accession number QKKF02033175.1) [22] and applied for promoter analysis. Two candidate transcription start sites (TSSs) were predicted at a distance from miR-263a of 385 bp and 3786 bp, which were named TSS1 and TSS2, respectively. The possible promoter regions located 1.4 kb upstream of each TSS were cloned and inserted into the pGL4.10 plasmid for promoter activity analysis. The relative luciferase activity driven by the 1.4 kb sequence upstream of TSS1 was nearly 10-fold that of the negative control group transfected with empty pGL4.10 in human 293T cells, while the 1.4 kb sequence upstream of TSS2 did not exhibit obvious promoter activity (Fig 1B). The transcript amplified from TSS1 was obtained via RT–PCR from the nuclear fraction, whereas no transcripts were observed when the amplification started 96 bp upstream of TSS1 (Fig 1C and 1D). These results demonstrated that the 1.4 kb sequence upstream of TSS1 contained the promoter of miR-263a and that TSS1 could be the transcription start site.

### Transcription factor YY1 inhibits miR-263a transcription

**Fig 2:**
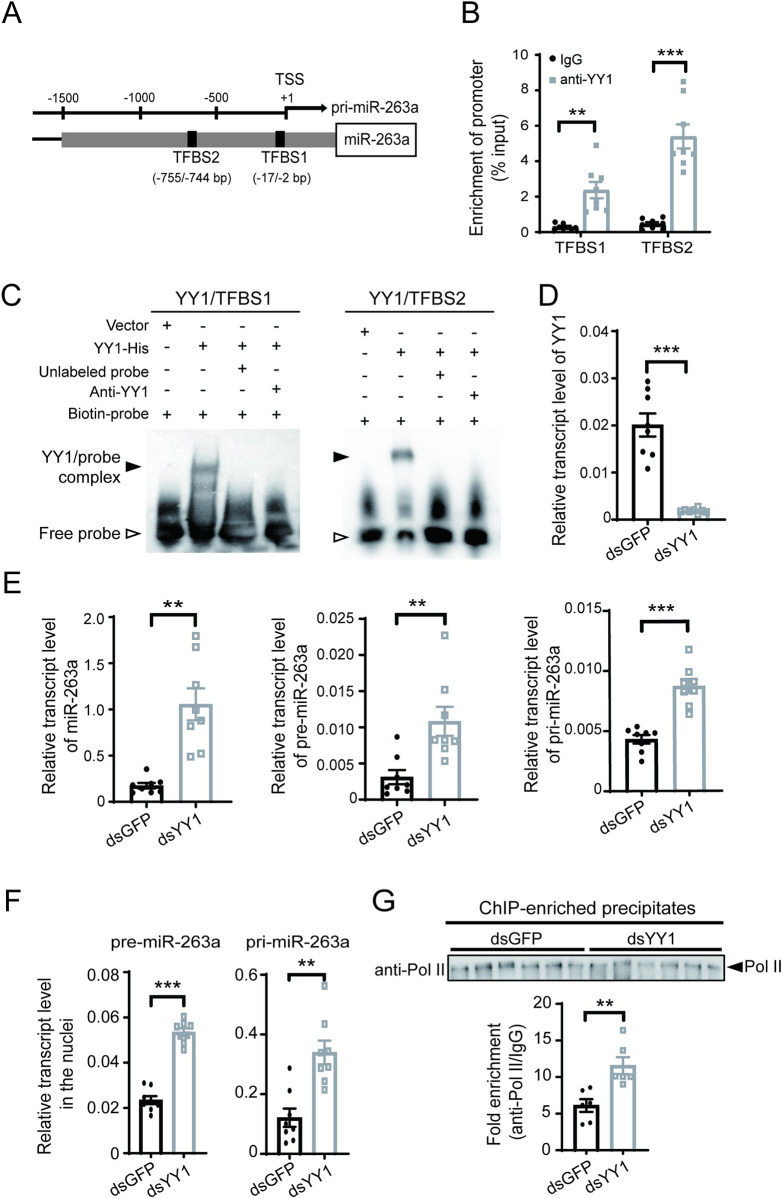
Transcription factor YY1 inhibits miR-263a transcription. (A) Schematic diagram showing two putative YY1 binding sites, TFBS1 and TFBS2 (black rectangles), in the promoter region of miR-263a. The transcription start site (TSS) is designated position +1. pri-miR-263a, primary miR-263a. (B) Relative enrichment of the two binding sites of YY1 measured by ChIP-qPCR (n = 8). IgG was used as the negative control. (C) EMSA assay on the binding of recombinant YY1-His to the DNA sequences containing TFBS1 (left image) or TFBS2 (right image) using the biotin-labeled probes. (D) Relative transcription level of *YY1* in the nonviruliferous planthoppers after injection with dsRNAs for *YY1* (dsYY1) or *GFP* (dsGFP) for 3 d (n = 8). (E) Relative transcript levels of miR-263a, precursor miR-263a (pre-miR-263a) and pri-miR-263a in planthopper whole bodies after injection with dsYY1 or dsGFP (n = 8). (F) Relative transcript levels of pre-miR-263a and pri-miR-263a in the nuclear extracts after injection with dsYY1 or dsGFP (n = 8). (G) Relative enrichment of the putative RNA polymerase II (Pol II) binding sequence precipitated by the anti-Pol II monoclonal antibody to that precipitated by IgG in the dsYY1- or dsGFP-injected nonviruliferous planthopper measured by ChIP-qPCR (n = 6). Comparable amounts of Pol II in the two groups were detected by western blot. Data information: Graphs show mean values and standard errors. For (B), (D), (E), (F), and (G) comparison was analyzed by Student’s t test. **, P<0.01. ***, P<0.001.

Transcription factors that possibly bind to the 1.4 kb promoter region of miR-263a were predicted. Among the nine putative transcription factors (S1 Table), YY1 has been reported to target the promoter regions of many miRNAs in mammalian cells [23, 24] and interact with RSV NPs in the nuclei of planthopper cells [25]. Thus, we focused on YY1 to study its regulation of miR-263a transcription. Two putative YY1 binding sites, namely, TFBS1 (TCACCGTCACAGCCAC) and TFBS2 (GAAGATGGAATT), were identified from -2 to -17 bp and from -744 to -755 bp upstream of the TSS, respectively (Fig 2A). Owing to the high sequence identity between YY1 homologs of human and small brown planthopper [25], the anti-human YY1 polyclonal antibody could specifically recognize planthopper YY1 (S1A Fig). Chromatin immunoprecipitation combined with quantitative real-time PCR (ChIP-qPCR) using the human anti-YY1 polyclonal antibody showed that both TFBS1 and TFBS2 were enriched in the YY1 immunoprecipitates relative to the IgG control (Fig 2B), indicating that the two sites were targets of YY1 in the promoter of miR-263a. In the electrophoretic mobility shift assay (EMSA) analysis, the recombinant YY1-His caused a significant mobility shift of the DNA sequences containing TFBS1 or TFBS2, which were recognized by the biotin-labeled probes. The addition of unlabeled probes or anti-YY1 antibodies reduced the amount of the shifted DNA sequences (Fig 2C). The EMSA results further confirmed the binding of YY1 to the promoter of miR-263a.

When silencing the expression of *YY1* in nonviruliferous planthoppers for 3 d after the injection of double-stranded RNA (dsRNA) targeting *YY1* (dsYY1-RNA) (Fig 2D), the amounts of miR-263a, precursor miR-263a and primary miR-263a significantly increased compared with that of the control group, which was injected with dsGFP-RNA (Fig 2E). Moreover, the transcript levels of primary miR-263a and precursor miR-263a in the nuclei were also upregulated based on the interference of *YY1* expression (Fig 2F). These results indicated that YY1 had an inhibitory effect on miR-263a transcription.

Primary miRNAs are transcribed by RNA polymerase II (Pol II), and the Pol II binding site is usually located within 100 bp upstream of the TSS [26]. Because the YY1 binding sites TFBS1 and TFBS2 are close to the Pol II binding site, YY1 may affect the interaction of Pol II with the promoter region of miR-263a. To verify this hypothesis, ChIP-qPCR was applied to evaluate the effect of YY1 on the binding of Pol II to the promoter of miR-263a using a human anti-Pol II monoclonal antibody, which recognizes the largest catalytic subunit RPB1. Human RPB1 (registration number NP_000928.1) has 70% identity to the small brown planthopper RPB1 (RZF37892.1), which is 183.8 kDa. Therefore, the human anti-Pol II antibody was capable of pulling down planthopper Pol II (Figs 2F and S1B and S2A). After injecting ds*YY1*-RNA to knock down the expression of *YY1* for 3 d in nonviruliferous planthoppers, the amount of putative Pol II binding sequence (nearly 100 bp upstream of TSS) in the Pol II-immunoprecipitates significantly increased in contrast to the ds*GFP*-RNA injection group (Fig 2G). These results suggested that YY1 weakened the promoter binding activity of Pol II to suppress miR-263a transcription.

### Binding with RSV NP promotes the inhibitory effect of YY1 on miR-263a transcription

**Fig 3:**
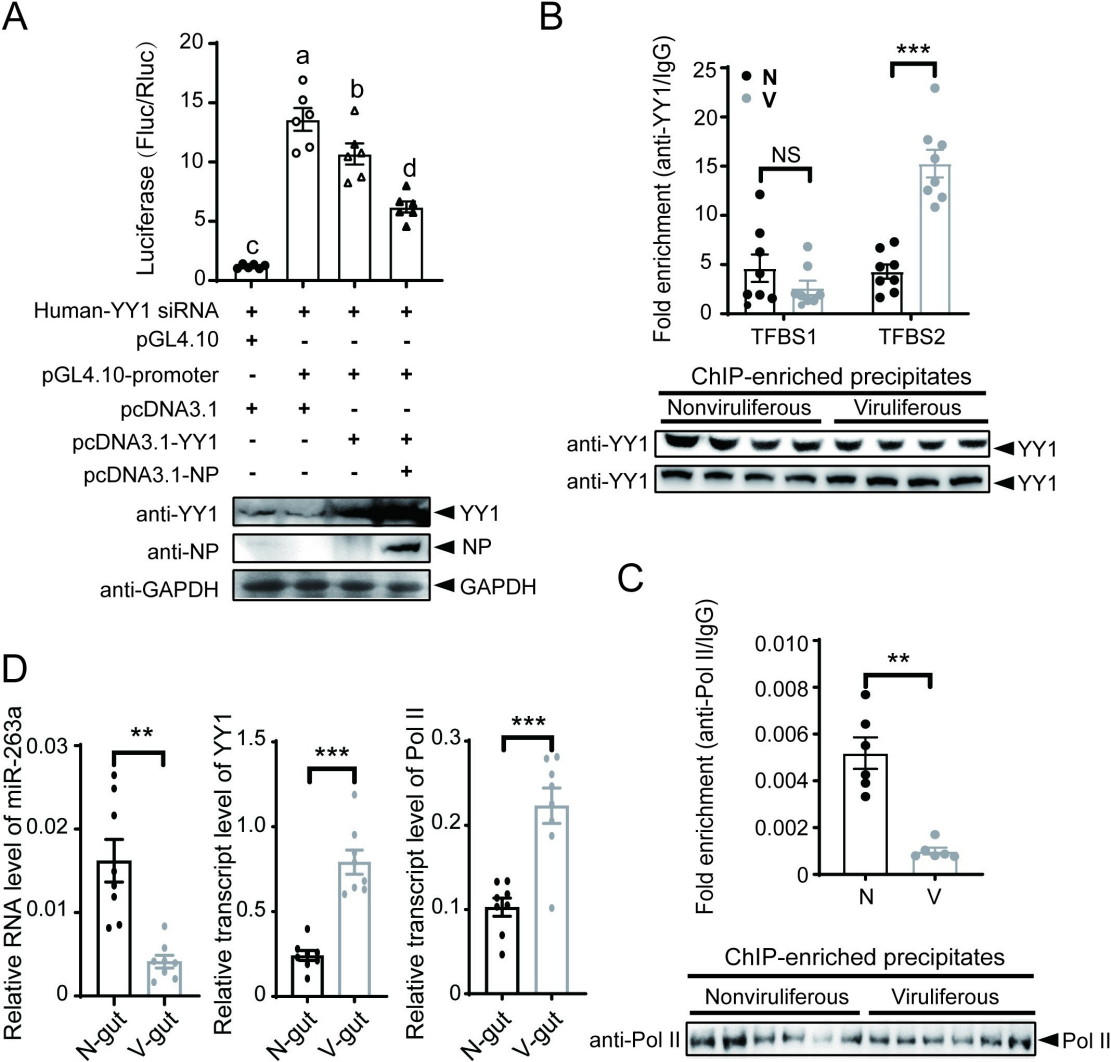
Binding with RSV NP promotes the inhibitory effect of YY1 on miR-263a transcription. (A) Dual luciferase reporter assay on the promoter activity of miR-263a after expression of planthopper YY1 or both YY1 and RSV NP in 293T cells with the addition of siYY1. The relative activity of firefly luciferase (Fluc) to *Renilla* luciferase (Rluc) is presented. Empty pGL4.10 and pcDNA3.1 were used in the negative control groups. Western blot assays show the protein expression. Different letters indicate significant differences in Tukey’s multiple comparison test. (B) Enrichment of YY1 binding sites TFBS1 and TFBS2 precipitated by the anti-YY1 polyclonal antibody relative to that precipitated by IgG in nonviruliferous (N) and viruliferous (V) planthoppers measured by ChIP-qPCR (n = 8). Comparable amounts of YY1 in the two groups were detected by western blot. (C) Enrichment of the putative RNA polymerase II (Pol II) binding sequence precipitated by the anti-Pol II monoclonal antibody relative to that precipitated by IgG in N and V planthoppers measured by ChIP-qPCR (n = 6). Comparable amounts of Pol II in the two groups were detected by western blot. (D) Relative transcript levels of miR-263a, *YY1* and *Pol II* in the guts of nonviruliferous and viruliferous planthoppers. Data information: Graphs show mean values and standard errors. NS, no significant difference. **, *P* < 0.01. ***, *P* < 0.001.

Considering the binding of RSV NPs with YY1 [25], we explored the influence of the two protein interactions on the ability of YY1 to negatively regulate miR-263a transcription. Planthopper YY1 was expressed alone and with NP in human 293T cells that were transfected with recombinant pGL4.10 plasmid containing the 1.4 kb promoter sequence of miR-263a. Due to the high identity between human and planthopper YY1 homologous proteins [25], 293T cells were treated with siRNA targeting human *YY1* to reduce its influence (S3 Fig). The expression of planthopper YY1 alone inhibited the relative luciferase activity driven by the promoter of miR-263a, and the extent of inhibition was elevated significantly when both YY1 and NP were expressed (Fig 3A).

Compared to the nonviruliferous planthoppers, more TFBS2 sequences (but not TFBS1 sequences) were immunoprecipitated using the anti-YY1 polyclonal antibody from the viruliferous planthoppers by ChIP-qPCR (Figs 3B and S2B). At the same time, fewer putative Pol II binding sequences in the promoter of miR-263a were immunoprecipitated with the human anti-Pol II monoclonal antibody from the viruliferous planthoppers (Figs 3C and S2C). In the gut of viruliferous planthoppers, miR-263a was downregulated while *YY1* and *Pol II* were both upregulated compared to nonviruliferous planthoppers (Fig 3D). These results indicated that RSV NP promoted the inhibitory effect of YY1 on miR-263a transcription by enhancing YY1 binding and reducing Pol II binding to the promoter of miR-263a.

### Identification of RSV vsRNAs putatively targeting primary miR-263a

**Fig 4:**
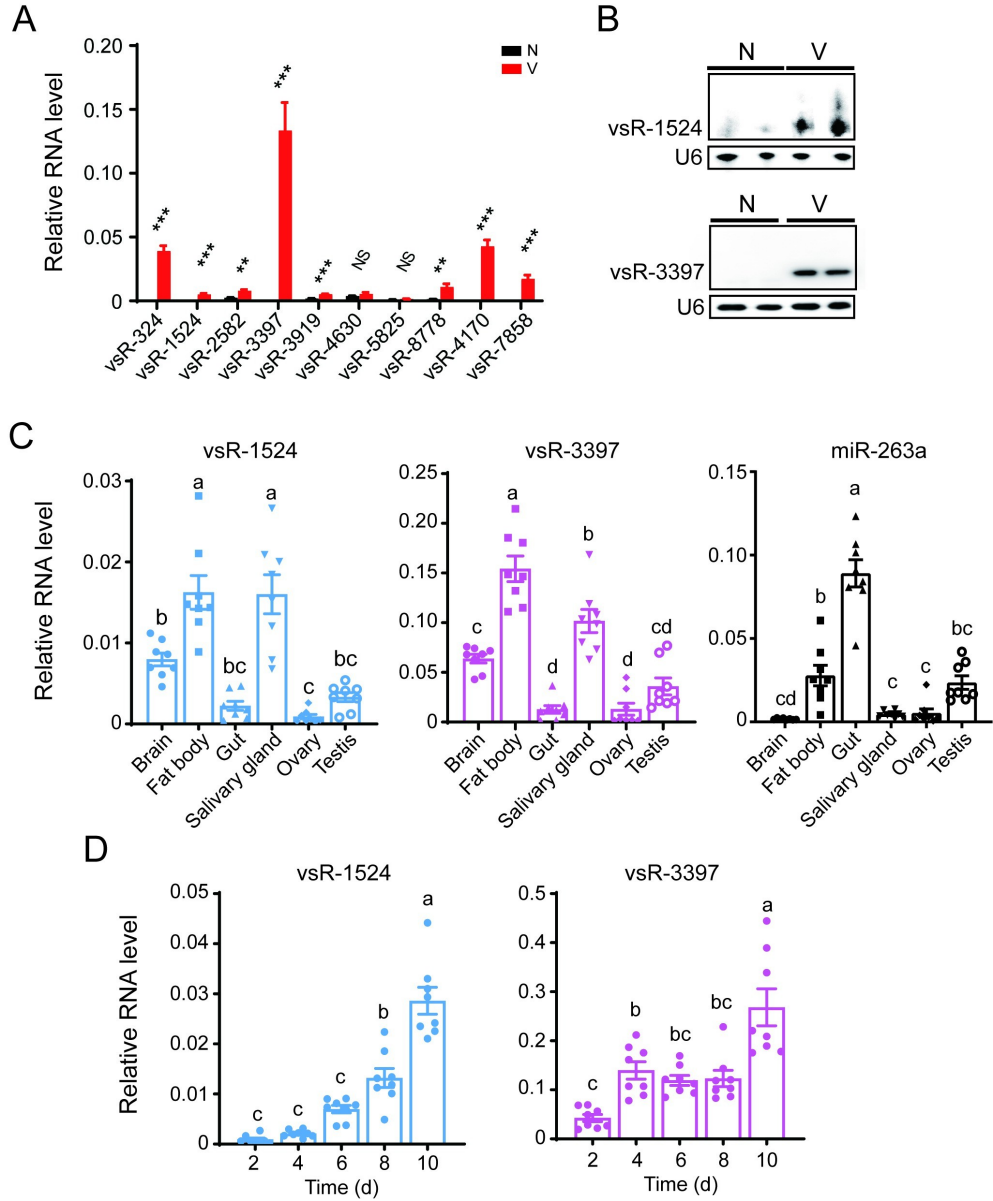
Identification of RSV vsRNAs putatively targeting primary miR-263a. (A) Relative RNA levels of the top ten most abundant vsRNAs in nonviruliferous (N) and viruliferous (V) fourth-instar planthoppers (n = 8). NS, no significant difference. **, P<0.01. ***, P<0.001. (B) Identification of vsR-1524 and vsR-3397 in N and V planthoppers by northern blot using biotin-labeled LNA oligonucleotide probes. (C) and (D) Relative RNA levels of vsR-1524, vsR-3397, and miR-263a in six organs of viruliferous adult planthoppers (C) and in the planthoppers at different days post-injection with RSV crude extractions (D) (n = 8). Different letters indicate significant differences in Tukey’s multiple comparison test. Planthopper U6 snRNA was used as an endogenous control for vsRNA in each experiment. Data information: Graphs show mean values and standard errors.

In addition to the seven proteins, RSV generates vsRNAs in small brown planthoppers [14]. No typical stem–loop secondary structure was predicted for the RSV vsRNAs, suggesting that these vsRNAs did not belong to miRNAs. However, no perfectly complementary sites for RSV vsRNAs were found in the primary miR-263a and its 2 kb upstream sequences. In addition to binding in the canonical perfectly complementary manner, siRNAs can bind targets in a miRNA-silencing manner through partial sequence complementarity [27]. Therefore, we predicted possible targets of RSV vsRNAs in primary miR-263a and its 2 kb upstream sequences via miRNA silencing using two methods. In total, 25 candidate vsRNAs were selected by both methods (S2 Table), and the top ten most abundant vsRNAs were selected for verification. qPCR showed that five putative vsRNAs, namely, vsR-324, vsR-1524, vsR-3397, vsR-4170, and vsR-7858, were detected in viruliferous but not nonviruliferous planthoppers (Fig 4A). Sanger sequencing demonstrated that only vsR-1524 (5’-AGGATGTGTTGGTCTCTAGCT-3’) and vsR-3397 (5’-CATCGTCTGTGGGTTCTGTGGA-3’) had right sequences of 21 nt and 22 nt, respectively. The presence of the two vsRNAs in viruliferous planthoppers was further verified using specific probes in northern blots (Fig 4B). These results supported the identity of vsR-1524 and vsR-3397 as RSV vsRNAs.

In viruliferous planthoppers, vsR-1524 and vsR-3397 showed a similar expression profile at the organ level, with the highest expression in the fat body and salivary gland and the lowest expression in the gut and reproductive organs (Fig 4C). miR-263a showed the highest expression in the gut and the lowest expression in the brain, salivary gland and ovary. Therefore, the expression trend of vsR-1524 and vsR-3397 was contrary to that of miR-263a especially in the gut and salivary gland (Fig 4C). When nonviruliferous planthoppers were infected with RSV for 2, 4, 6, 8, and 10 d, the expression of vsR-1524 and vsR-3397 increased with viral replication (Fig 4D).

### vsR-3397 suppresses miR-263a transcription by targeting the promoter region

**Fig 5:**
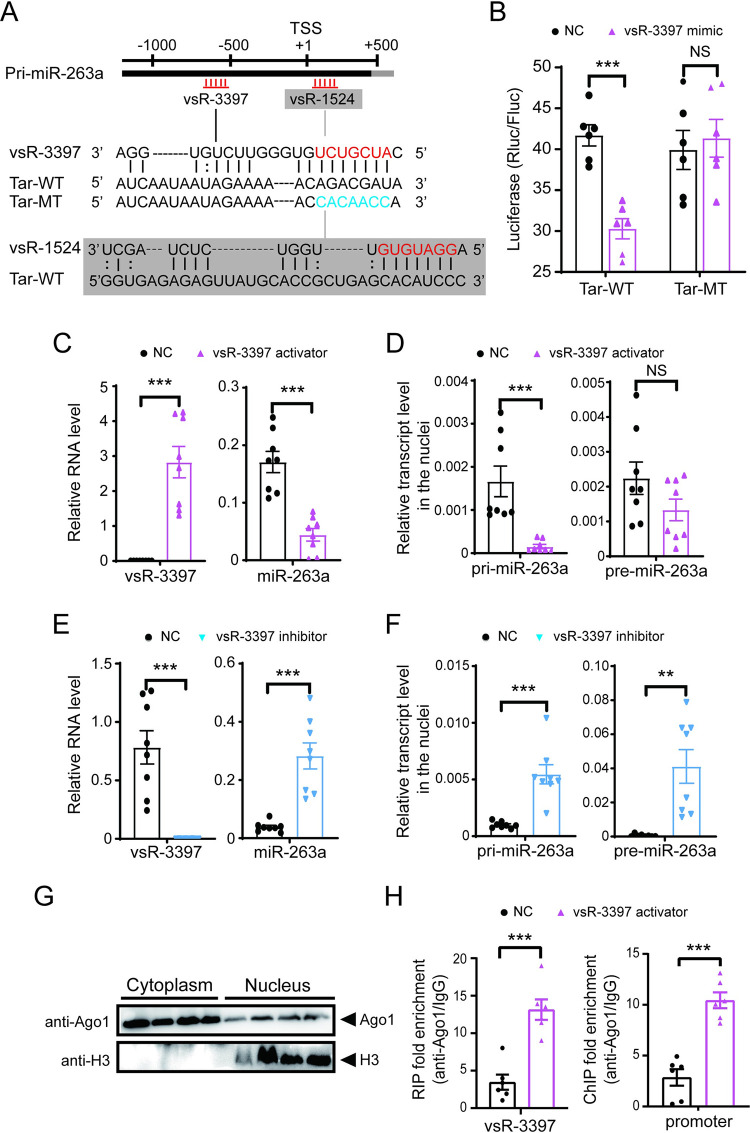
vsR-3397 suppresses miR-263a transcription by targeting the promoter region. (A) Sequence alignment of vsR-3397 and vsR-1524 with their predicted target sites (Tar-WT) in the promoter region of miR-263a. The seed sequences are in red. Mutated nucleotides in the target (Tar-MT) are in blue. TSS, transcription start site. pri-miR-263a, primary miR-263a. (B) Dual luciferase reporter assays in S2 cells cotransfected with recombinant psiCHECK2 plasmids containing Tar-WT or Tar-MT of vsR-3397 and 100 nM vsR-3397 mimics (n = 6). (C) and (D) Relative RNA levels of vsR-3397 and miR-263a in the whole body (C) and relative transcript levels of pri-miR-263a and precursor miR-263a (pre-miR-263a) in the nuclei (D) of nonviruliferous planthoppers after injection with the vsR-3397 activator for 4 d (n = 8). (E) and (F) Relative RNA levels of vsR-3397 and miR-263a in the whole body (E) and relative transcript levels of pri- and pre-miR-263a in the nuclei (F) of viruliferous planthoppers after injection with the vsR-3397 inhibitor for 4 d (n = 8). (G) Western blot analysis of Ago1 in the nuclear and cytoplasmic extracts from viruliferous planthoppers. (H) Relative enrichment of vsR-3397 and its promoter region in the nucleus fraction of viruliferous planthoppers measured by RIP and ChIP combined with qPCR after injection with vsR-3397 activator for 4 d (n = 6). Mouse IgG instead of the anti-Ago1 monoclonal antibody was used as a negative control. Data information: Graphs show mean values and standard errors. NS, no significant difference. *, P<0.05. **, P<0.01. ***, P<0.001.

The predicted target site for vsR-1524 or vsR-3397 was from 68 to 94 bp downstream of the TSS of primary miR-263a or from -573 to -597 bp upstream of the TSS in the promoter region (Fig 5A). The seed region from 2 to 8 nt of the two vsRNAs perfectly matched the target sequences (Fig 5A). Direct interactions between the two vsRNAs and their putative target sites were verified using dual luciferase assays in *Drosophila* S2 cells. Relative luciferase activities in cells transfected with 233 bp of sequences containing the putative target site significantly decreased in the presence of vsR-3397 at 10 or 100 nM (S4A Fig). Mutations of 7 bp at the site of the target corresponding to the seed region of vsR-3397 abolished the ability of vsR-3397 to suppress enzymatic activity (Fig 5B). In contrast, relative luciferase activities in cells transfected with 190 bp of sequences containing the putative target sequence were not affected by the presence of vsR-1524 at 1, 10, or 100 nM (S4B Fig). This experiment showed that the promoter region of miR-263a from -573 to -597 bp was targeted by vsR-3397.

The regulation of vsR-3397 on miR-263a transcription was further explored. When the synthetic activator of vsR3-3397 was injected into nonviruliferous planthoppers for 4 d, the level of miR-263a dropped down (Fig 5C), and the transcript level of primary miR-263a also significantly decreased in the nuclei compared to the injection of a control activator, while the precursor miR-263a only showed a declining trend in the nuclei (Fig 5D). Moreover, when the inhibitor, i.e., the complementary sequence of vsR3-3397, was injected into viruliferous planthoppers for 4 d, the amount of miR-263a (Fig 5E) and transcript levels of primary miR-263a and precursor miR-263a in the nuclei were upregulated compared to the injection of a control inhibitor (Fig 5F). When the nuclear fraction was isolated, we found the existence of Ago1 in the nuclei (Fig 5G). RNA immunoprecipitation combined with qPCR (RIP-qPCR) and ChIP-qPCR assays using an anti-Ago1 monoclonal antibody [28] showed that vsR-3397 and its promoter region were enriched in the Ago1-immunoprecipitation from the nuclear fraction of viruliferous planthoppers after injection with vsR-3397 activator for 4 d compared to the IgG pull-down group (Fig 5H). These results demonstrated that vsR-3397 negatively regulated miR-263a transcription in the nuclei.

### vsR-3397 inhibits RSV replication by affecting miR-263a

**Fig 6:**
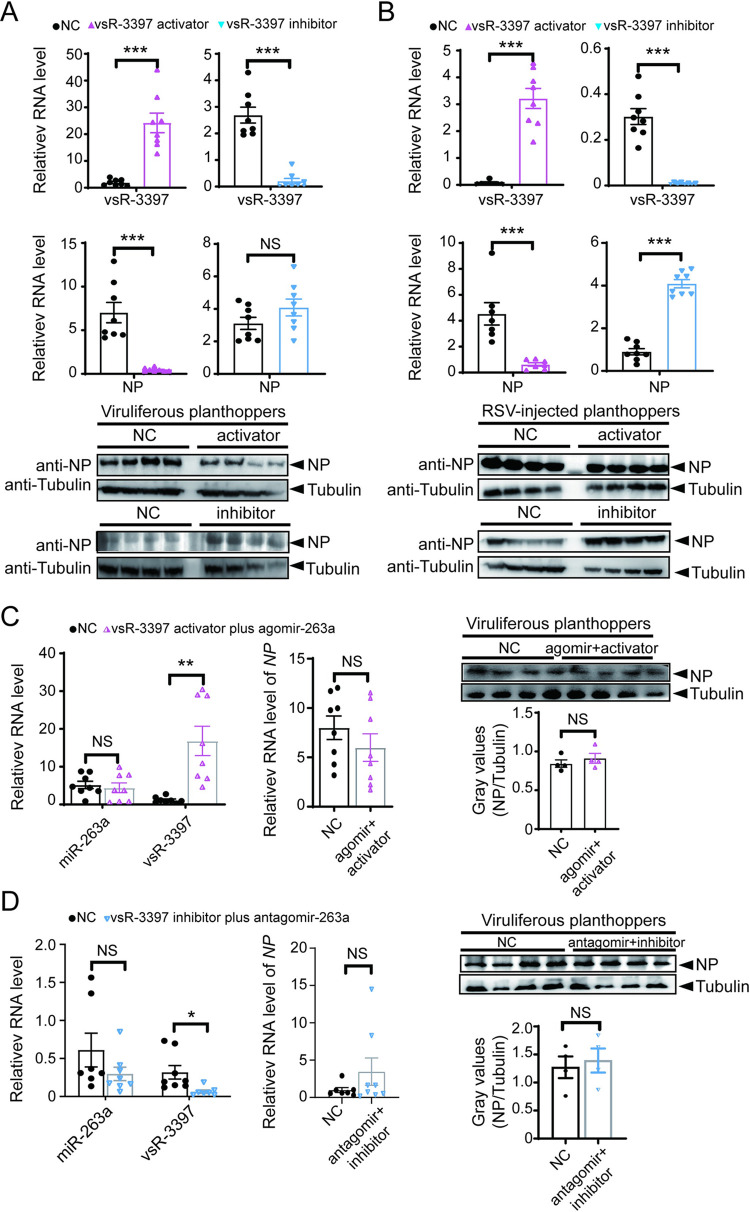
vsR-3397 inhibits RSV replication by affecting miR-263a. (A) and (B) Relative RNA levels of vsR-3397 and RSV *NP* (n = 8) and the protein level of NP (n = 4) in the viruliferous planthoppers after injection with the vsR-3397 activator or inhibitor for 4 d (A) and in the nonviruliferous planthoppers after injection with the mixture of RSV and the activator or inhibitor of vsR-3397 for 6 d (B). (C) and (D) Relative RNA levels of miR-263a, vsR-3397 and RSV *NP* (n = 8) and protein level of NP (n = 4) in the viruliferous planthoppers after injection with the mixture of vsR-3397 activator and miR-263a agomir (C) or vsR-3397 inhibitor and miR-263a antagomir (D) for 4 d. NC, negative control. The NC sequences for vsRNA activator and miR-263a agomir are 5’-UUCUCCGAACGUGUCACGUTT-3’ (sense) and 5’-ACGUGACACGUUCGGAGAATT-3’ (antisense). The NC sequence for vsRNA inhibitor and miR-263a antagomir is 5’-ACGUGACACGUUCGGAGAATT-3’. Data information: Graphs show mean values and standard errors. NS, no significant difference. *, *P* < 0.05. **, *P* < 0.01. ***, *P* < 0.001.

The effect of vsR-3397 on RSV replication was investigated based on injecting the activator or inhibitor of vsR-3397. In viruliferous planthoppers, the vsR-3397 activator significantly reduced the RSV load in terms of the RNA and protein levels of viral *NP*, although the vsR-3397 inhibitor did not produce an obvious influence on the viral load at 4 d post injection (dpi) (Figs 6A and S5A). When nonviruliferous nymphs were injected with a mixture of RSV and the activator or inhibitor of vsR-3397, the RSV load significantly decreased in the presence of the activator and increased with the inhibitor at 6 dpi (Figs 6B and S5B). Therefore, vsR-3397 produced an antiviral effect on RSV. However, when vsR-3397 activator and miR-263a agomir were injected together in viruliferous planthoppers, the amount of vsR-3397 was well-promoted, the level of miR-263a remained unchanged, and the RSV load no longer changed (Fig 6C). On the other hand, injection of both vsR-3397 inhibitor and miR-263a antagomir in viruliferous planthoppers did not affect RSV accumulation (Fig 6D). These findings indicated that vsR-3397 inhibited RSV replication by affecting miR-263a.

## Discussion

**Fig 7:**
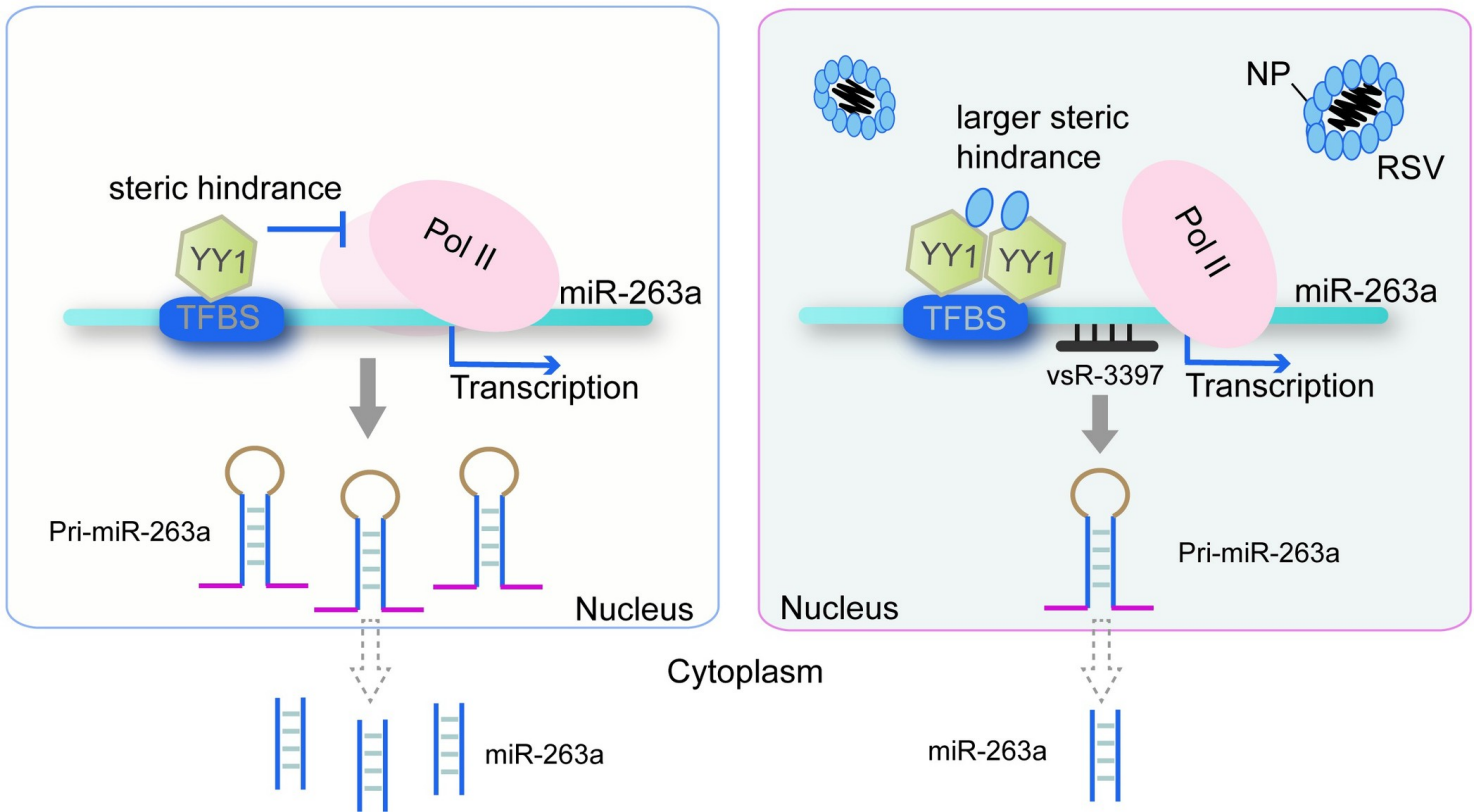
Model of the regulatory mechanisms for miR-263a by RSV. The NP of RSV binds the transcription factor YY1 to promote its inhibitory effect on miR-263a transcription probably due to enlargement of the steric hindrance to the binding of Pol II to the promoter with more YY1. At the same time, RSV-derived small RNA, vsR-3397, directly targets the promoter region of miR-263a to downregulate miR-263a transcription. TFBS, transcription factor binding site.

The sustainable infection of viruses in their insect vectors depends on the balance between the virus load and insect immune system. An endogenous miRNA of the small brown planthopper, miR-263a, which is beneficial for RSV replication, is downregulated upon viral infection. In the present study, we disclosed the mechanisms of this inhibitory effect on miR-263a expression from RSV, which utilized viral small RNA vsR-3397 that directly bound the promoter of miR-263a and the nucleocapsid protein that enhanced the activity of the miR-263a transcription factor YY1 (Fig 7).

Regulation of vsR-3397 to miR-263a expression through binding to the miRNA promoter is a new mechanism. Various functional mechanisms have been reported for viruses regulating the expression of host miRNAs. For example, induction of human miR-155 by Epstein–Barr virus depends on multiple cell signaling pathways, including the transcription factor AP-1 family member-mediated activation of miR-155 transcription [29]. West Nile virus (WNV) and dengue virus (DENV) sequester dsRNA binding proteins (Dicer proteins) to inhibit miRNA processing [30]. Tomato bushy stunt virus inhibits the maturation of host miRNAs by suppressing miRNA-related RISC loading activity [31]. Human cytomegalovirus (HCMV) accelerates host miR-17 family degradation via a virus-derived nc-transcript, which contains binding sites for miR-17 members [32]. In our work, RSV-derived vsR-3397 inhibited insect miR-263a expression by directly binding to the promoter region of miR-263a, leading to RSV replication suppression. Whether the binding of vsR-3397 induces degradation of the promoter region requires further exploration. uld mainly result from the function of vsR-3397 on miR-263a.

vsR-3397 acts on targets in a miRNA-silencing manner, albeit without a classical stem–loop structure of precursor miRNAs. RSV-derived small RNAs did not seem to be miRNAs because no stem–loop secondary structure was predicted for these vsRNAs. Some RNA viruses can produce miRNAs. For instance, KUN-miR-1 and DENV-vsRNA-5 are miRNAs derived from the 3’ terminal stem–loop regions of WNV and DENV genomes but produce opposite effects on viral replication [33]. The sequence of vsR-3397 was not perfectly complementary to the target site of the miR-263a promoter. Instead, the seed region from 2 to 8 nt of vsR-3397 matched the target sequences, which was similar to the mode of action of miRNAs in animals [34]. A similar phenomenon was found in nonvirus-derived siRNAs. In human HeLa cells, gene expression profiling revealed that siRNAs may silence nontargeted genes that share only 11 contiguous nucleotides of identity with these siRNAs [35]. A human siRNA can work on the target sequence of *CXCR4*, which has a central bulge or mismatches corresponding to the 9^th^ to 11^th^ positions of the siRNA and results in the repression of reporter gene expression *in vitro* [36,37].

The nucleocapsid protein NP of RSV strengthened the extent to which YY1 suppressed miR-263a transcription. YY1 is a ubiquitously expressed transcription factor that not only interacts with DNAs to regulate gene transcription [38] but also functions as an upstream regulator of miRNAs in mammalian cells [39]. For example, YY1 directly targeted the enhancer sequence of primary miR-181a to induce its expression [40]. YY1 was also revealed to negatively regulate miR-500-5p expression by binding its promoter region [41]. In the present work, we found that YY1 worked as a negatively regulating factor for miR-263a expression in planthoppers by affecting the binding of Pol II to the promoter. The interaction of RSV NP with YY1 strengthened this suppression, probably due to enlargement of the steric hindrance to the binding of Pol II to the promoter of miR-263a with more YY1. Furthermore, NP only influences the interaction between YY1 and TFBS2, but not YY1 and TFBS1 in miR-263a promoter region. TFBS1 is localized from -2 to -17 bp and TFBS2 is from -744 to -755 bp upstream of the TSS of miR-263a. Obviously, TFBS1 is closer to TSS than TFBS2. It is generally believed that the core promoter, ranging from -50 bp upstream and +50 bp downstream of TSS, serves as a binding platform for recruiting the transcription machinery consisting of Pol II and its associated transcription factors [42,43]. Transcription can also be further suppressed or activated by silencers or enhancers, which are located distally from TSS and associated with transcription factors and transcription cofactors [44]. For our work, TFBS1 is within the core promoter and it is hard for NP to interrupt the transcription machinery and influence the interaction of YY1 and TFBS1. TFBS2, far away from TSS, functions like a regulatory element and NP works as a transcription cofactor to fine-tune miR-263a expression.

Our previous work revealed that YY1 positively regulated the transcription of *FAIM* and that the interaction of RSV NP with YY1 in nuclei inhibited *FAIM* transcription to activate antiviral apoptotic reactions [25]. This phenomenon is contrary to the present work, probably resulting from the complicate functions of YY1, which can repress and activate gene transcription. The repression of gene transcription is fulfilled by the competition of YY1 with activator proteins in overlapping promoter binding sites [45] or the recruitment of corepressors that directly act to facilitate transcriptional repression [46]. In our study, YY1 weakens the promoter binding activity of Pol II to suppress miR-263a transcription. In the contrast, YY1 activates gene transcription by recruitment of coactivators [47]. Moreover, YY1 sometimes performs activation and repression on the same promoter. For example, YY1 represses the adenovirus P5 promoter but it is converted into a transcriptional activator in the presence of adenovirus E1A protein [48]. Such complicate functions of YY1 are performed by different protein domains. The N-terminal amino acids 16–29 and 80–100 are within YY1 transcriptional activation domain and the zinc fingers near the C-terminus lie within YY1 transcriptional repression domain [49]. Activation of *FAIM* transcription and inhibition of miR-263a transcription may be mediated by the different domains of YY1 and NP binding could affect YY1’s function in different mechanisms.

In conclusion, we identified a new mechanism by which RSV regulates endogenous miRNAs in insect vectors, especially the crosstalk between virus-derived small RNAs and insect miRNAs. This ingenious adaptation strategy of phytoviruses prevents overreplication of viruses and thus ensures a harmonious relationship with the insect vectors to achieve efficient transmission in nature.

## Materials and methods

### Planthopper strains

Viruliferous and nonviruliferous small brown planthopper strains were incubated separately on rice (*Oryza sativa* Wuyujing) in glass incubators and screened using dot enzyme-linked immunosorbent assay (dot-ELISA) with the homemade monoclonal anti-NP antibody every three months [13].

### Bioinformatic analyses of the promoters, transcription factors and vsRNA binding sites of miR-263a

Promoters and TSSs of primary miR-263a were analyzed in the Promoter 2.0 Prediction Server (https://services.healthtech.dtu.dk/service.php?Promoter-2.0). Transcription factors of primary miR-263a were predicted in AnimalTFDB (v3.0) (http://bioinfo.life.hust.edu.cn/AnimalTFDB/#!/). The typical stem–loop secondary structure of RSV vsRNAs was analyzed using miRDeep2 [50]. The potential binding sites of RSV vsRNAs in the primary miR-263a and its 2 kb upstream sequences were predicted using miRanda [51] and RNAhybrid [52] algorithms with cutoff values of -18 and -25 kcal mol^−1^ for the minimum free energy (MFE) of the RNA duplex.

### Promoter luciferase report assay

The 1.5 kb sequences at ~1.4 kb upstream and 100 bp downstream of TSS1 or TSS2 of miR-263a were amplified and subcloned into the pGL4.10 vector, which had a *firefly luciferase* (*Fluc*) gene (Thermo Fisher Scientific, Waltham, MA, USA). The full-length open reading frames (ORFs) of *L*. *striatellus YY1* (Registration number in GenBank MT113358) and RSV *NP* (DQ299151) were cloned and inserted into the pcDNA3.1 vector (Invitrogen, Carlsbad, CA, USA). siRNAs for human *YY1* (NM_003403) were synthesized by the Huigene Biotechnology Company (Beijing, China). The pGL4.13 plasmid (Promega, Madison, WI, USA) was used as a positive control. The pRL-TK plasmid containing a *Renilla luciferase* (*Rluc*) gene (Promega) was used as a reference to normalize the transfection efficiency. Plasmids were transfected into 293T cells using Lipo3000 (Invitrogen). After 24 h at 37°C, the cells were collected and analyzed using the Dual-Glo Luciferase Assay System (Promega) in a luminometer (Promega). Six replicates were prepared for each group. The relative activity of Fluc normalized to Rluc activity is presented as the mean ± SE. The primers used in this experiment are listed in S3 Table.

### vsRNA target validation

DNA was extracted from small brown planthoppers using Puregene Core Kit A (Qiagen Sciences, Maryland, USA) following the manufacturer’s instructions. The 189 bp or 232 bp sequence containing the predicted target sites on the miR-263a coding gene for vsR-1524 or vsR-3397 was cloned from the DNA template and inserted into the psiCHECK2 plasmid (Promega). Site mutations in the sequences complementary to the “seed” sites of vsRNAs were performed with a KOD-Plus mutagenesis kit (Toyobo Bio-Technology). The mimics were double-stranded RNAs of vsR-1524 and vsR-3397. The sequences of the negative control (NC) for vsRNA activators were 5’-UUCUCCGAACGUGUCACGUTT-3’ (sense) and 5’-ACGUGACACGUUCGGAGAATT-3’ (antisense). The mimics and NC were synthesized by Huigene Biotechnology Company. S2 cells were cotransfected with recombinant psiCHECK2 plasmids and various concentrations (1 nM, 10 nM, and 100 nM) of vsRNA mimics or NC using Lipofectamine 3000 (Invitrogen). After transfection for 24 h at 28°C, cells were collected for the luciferase assay as described above. Six replicates were prepared for each group. The relative activity of Rluc normalized to Fluc activity is presented as the mean ± SE. The primers used in this experiment are listed in S3 Table.

### Separation of nuclear and cytoplasmic fractions

To identify miR-263a promoter region and evaluate the levels of pri-miR-263a and pre-miR-263a, nuclear and cytoplasmic fractions were extracted from viruliferous planthoppers using the Nuclear and Cytoplasmic Extraction kit (BestBio, Shanghai, China) and validated by western blotting as previously described [25]. The reference proteins for nuclear and cytoplasmic proteins were histone H3 and GAPDH, which were detected using polyclonal anti-H3 antibody and anti-GAPDH antibody, respectively (Abcam, Cambridge, UK).

### RNA isolation and reverse transcription

Total RNA from 5 planthoppers and RNA from the nuclear fraction, 20 salivary glands, 10 brains, 8 guts, 8 fat bodies, 8 ovaries or 8 testes were extracted with TRIzol reagent (Invitrogen) following the manufacturer’s instructions. After eliminating DNA contamination by DNase (Promega), the quality of RNA was detected by a NanoDrop spectrophotometer (Thermo Fisher Scientific) and gel electrophoresis. Two micrograms of RNA were reverse transcribed to cDNA using the Superscript III First-Strand Synthesis System (Invitrogen) and random primers (Promega) [53]. For miRNAs or vsRNAs, 1 μg of RNA was reverse transcribed using the miRcute Plus miRNA First-Strand cDNA Synthesis Kit (Tiangen, Beijing, China) [21].

### Quantitative real-time PCR (qPCR) and reverse transcription-PCR (RT–PCR)

A LightCycler 480 SYBR Green I Master Mix (Roche, Basel, Switzerland) or miRcute miRNA qPCR Detection Kit (Tiangen) was used for qPCR amplification on a LightCycler 480 instrument II (Roche). Planthopper *EF2* and U6 snRNA were quantified as endogenous controls for mRNAs and small RNAs, respectively. Two primer pairs (F1/R1 and F2/R2) were designed to amplify transcripts starting exactly from TSS1 and at 96 bp upstream of TSS1 with the cDNA template of the nuclear fraction using RT–PCR as previously described [54]. Primers are listed in S3 Table. All PCR products were sequenced for validation.

### Northern blot

Total RNA was extracted from nonviruliferous and viruliferous planthoppers, separated on 15% (wt/vol) denaturing polyacrylamide gels, and electroblotted onto nylon membranes (Invitrogen). Digoxigenin (DIG)-labeled LNA oligonucleotide probes (20 ng/mL, RiboBio, Guangzhou, China) were generated for the antisense sequence of vsR-1524, vsR-3397, and U6 snRNA (5’-CTAATCTTCTCTGTATCGTTCC-3’). Probe hybridizations were performed at 43°C for vsR-3397 and 37°C for vsR-1524 or U6. The membranes were blocked with blocking buffer (Invitrogen) for 15 min followed by incubation with an anti-DIG monoclonal antibody (1:500, MBL, Nagoya, Japan) for 1 h and then with a HRP-linked anti-mouse IgG (1:500, Invitrogen) for 1 h. Detection was carried out using SuperSignal West Femto (Thermo Fisher Scientific).

### Western blot

Proteins from 293T cells, nuclear and cytoplasmic fractions, ChIP pulldown materials, or planthoppers treated with vsR-3397 inhibitor or activator were extracted using 1×PBS buffer (pH 7.2) and separated by sodium dodecyl sulfate–polyacrylamide gel electrophoresis (SDS–PAGE). Anti-tubulin polyclonal antibody (Abcam, Cambridge, UK), anti-YY1 polyclonal antibody (Thermo Fisher Scientific), anti-H3 polyclonal antibody (Abcam), anti-GAPDH polyclonal antibody anti-GAPDH (Abcam), homemade anti-NP monoclonal antibody [13], or RNA Pol II monoclonal antibody (Active Motif, Carlsbad, CA, USA) was applied in different experiments. The immunoblot signal was detected using SuperSignal West Femto (Thermo Fisher Scientific).

### Double-stranded RNA synthesis and delivery

dsRNAs for planthopper *YY1* and *GFP* were synthesized using the T7 RiboMAX Express RNAi System (Promega) as previously described [25]. A total of 23 nL of dsRNAs at 6 μg/μL was delivered into third-instar nymphs through microinjection using a Nanoliter 2000 instrument (World Precision Instruments, Sarasota, FL, USA). Primers are shown in S3 Table.

### Injection of vsRNA activator, inhibitor, miRNA agomir and antagomir

vsR-3397 inhibitor and miR-263a antagomir were chemically modified single-strand nucleotide sequences reverse complementary to vsR-3397 and miR-263a (Huigene). vsR-3397 activator and miR-263a agomir were chemically modified double-stranded vsR-3397 and miR-263a (Huigene). The NC sequences for vsRNA activator or miR-263a agomir were 5’-UUCUCCGAACGUGUCACGUTT-3’ (sense) and 5’-ACGUGACACGUUCGGAGAATT-3’ (antisense). The NC sequence for vsRNA inhibitor or miR-263a antagomir was 5’-ACGUGACACGUUCGGAGAATT-3’. A total of 13.8 nL of vsR-3397 activator, inhibitor, or NC at 200 μM, or vsR-3397 activator plus miR-263a agomir, vsR-3397 inhibitor plus miR-263a antagomir, or NC at 200 μM was delivered into viruliferous fourth-instar nymphs by microinjection using a Nanoliter 2000 system (World Precision Instruments). Planthoppers were collected for RNA or protein isolation four days after injection.

### Injection of RSV crude extractions

RSV crude extractions from viruliferous planthoppers were delivered into the hemolymph of nonviruliferous fourth-instar nymphs by microinjection through a glass needle using a Nanoliter 2000 system (World Precision Instruments) as previously described [25]. Insects were collected at 2, 4, 6, 8, and 10 dpi for qPCR assays.

### ChIP-qPCR analysis

ChIP assays were performed using a ChIP kit (BersinBio, Guangzhou, China) based on our previous study [25]. Immunoprecipitation was performed using normal rabbit/mouse IgG (Cell Signaling Technology), anti-YY1 polyclonal antibody (Thermo Fisher Scientific) [25], or anti-Pol II monoclonal antibody (Active Motif). The ChIP-enriched DNA fragment was subjected to qPCR analysis using a Light Cycler 480 II instrument (Roche). YY1-binding sites and the Pol II-binding region were detected via qPCR using specific primers (S3 Table). The results were analyzed via the percent input method as previously described [25].

### RIP-qPCR analysis

RIP assay was performed using RIP-Kit (BersinBio) as previously described [28]. Nuclear proteins were extracted from 40 fourth-instar viruliferous nymphs 4 d after the injection with vsR-3397 activator or NC and incubated with magnetic beads and 2 μg of a homemade anti-Ago1 monoclonal antibody [28] or normal mouse IgG (Abcam) at 4°C overnight. Total RNA was extracted by TRIzol reagent and reverse transcribed into cDNA using the miRcute Plus miRNA First-Strand cDNA Synthesis Kit (Tiangen) [28]. qPCR was performed to detect the RNA levels of vsR-3397. The RNA level of each target segment relative to that in the IgG control sample is reported as the mean ± SE. Student’s t-test was performed to evaluate the differences between the two means using SPSS 17.0.

### EMSA analysis

To determine the binding of YY1 to the miR-263a promoter, EMSA assay was performed using LightShift Chemiluminescent EMSA kit (Thermo Fisher Scientific). *YY1* cDNA was cloned into a pAc5.1B vector to express YY1-His fusion proteins in *Drosophila* S2 cells. S2 cells were transfected with pAc5.1B-YY1 using Lipofectamine 2000 (Invitrogen). Nuclear proteins were isolated at 24 h post transfection with a Nuclear and Cytoplasmic Extraction kit (BestBio). The biotin-labeled and unlabeled DNA probes (TFBS1: 5’-GTACGTTCACCGTCACAGCCACTCAAAGAA-3’; TFBS2: 5’-TGAAGTGGAAGATGGAATTAGGTTTG-3’) were obtained from Sangon Biotech (Shanghai, China). Nuclear protein extracts were incubated with 1 nM labeled probes in 20 μL binding reactions containing 2.5% Glycerol, 5 mM MgCl_2_, 50 ng/μL poly(dI/dC), and 0.05% NP-40. For competition studies, 50X molar excess of unlabeled probe or anti-YY antibody was pre-incubated with the nuclear extracts at 4°C for 1 h before binding reaction. The DNA-protein complex was analyzed in 4.5% native polyacrylamide gels and visualized using SuperSignal West Femto (Thermo Fisher Scientific).

### Statistical analysis

All data were analyzed using GraphPad Prism software. Error bars represented SEM. Six to eight replicates were prepared and assayed for each group of experiments. Data comparison between two groups was performed using an unpaired t test. Data comparisons among multiple groups were performed by one-way ANOVA with Tukey’s multiple comparison test.

## Supporting information

S1 Fig Verification of human anti-YY1 polyclonal antibody (A) and human anti-Pol II monoclonal antibody (B) in western blot analysis using the total proteins of small brown planthopper.
(TIF)

S2 Fig The complete images of western blots for ChIP analysis of Figs 2G (A), 3B (B) and 3C (C).
(TIF)

S3 Fig Relative transcript level of human *YY1* in 293T cells after transfection with human YY1 siRNAs (siYY1) (n = 6). *GFP* siRNAs were used in the negative control group (NC).
(TIF)

S4 Fig Dual luciferase reporter assays in *Drosophila* S2 cells cotransfected with recombinant psiCHECK2 plasmids containing the predicted target sequence of vsR-3397 or vsR-1524 and various concentrations of mimics of vsR-3397 (A) or vsR-1524 (B). The activity of *Renilla* luciferase (Rluc) relative to that of firefly luciferase (Fluc) is presented (n = 6). NC, negative control. Values were compared by Student’s t test. NS, no significant difference. **, P<0.01. ***, P<0.001.
(TIF)

S5 Fig Gray values showing the relative optical densities of NP to that of Tubulin. The relative optical densities of NP to that of Tubulin was calculated in the viruliferous planthoppers after injection with the vsR-3397 activator or inhibitor for 4 d (A), and in the nonviruliferous planthoppers after injection with the mixture of RSV and the activator or inhibitor of vsR-3397 for 6 d (B), corresponding to the western results of Fig 6A and 6B.
(TIF)

S1 Table Nine predicted transcription factors and their binding sites in the promoter region of miR-263a. (XLSX)

S2 Table Information on the 25 RSV candidate vsRNAs and their potential binding sites in primary miR-263a and its 2 kb upstream sequences. (XLSX)

S3 Table Sequences of the primers and probes used in the study. (DOCX)

## References

[1] Ding SW, Voinnet O. Antiviral immunity directed by small RNAs. Cell. 2007;130(3):413–26. Epub 2007/08/19. doi: 10.1016/j.cell.2007.07.039 ; PubMed Central PMCID: PMC2703654. 17693253 PMC2703654

[2] Kemp C, Imler JL. Antiviral immunity in drosophila. Curr Opin Immunol. 2009;21(1):3–9. Epub 2009/02/19. doi: 10.1016/j.coi.2009.01.007 ; PubMed Central PMCID: PMC2709802. 19223163 PMC2709802

[3] Blair CD, Olson KE. The role of RNA interference (RNAi) in arbovirus-vector interactions. Viruses. 2015;7(2):820–43. Epub 2015/02/19. doi: 10.3390/v7020820 ; PubMed Central PMCID: PMC4353918. 25690800 PMC4353918

[4] Kutter C, Svoboda P. miRNA, siRNA, piRNA: Knowns of the unknown. RNA Biol. 2008;5(4):181–8. Epub 2009/02/03. doi: 10.4161/rna.7227 . 19182524

[5] Sodroski C, Lowey B, Hertz L, Jake Liang T, Li Q. MicroRNA-135a modulates Hepatitis C virus genome replication through downregulation of host antiviral factors. Virol Sin. 2019;34(2):197–210. Epub 2018/11/21. doi: 10.1007/s12250-018-0055-9 ; PubMed Central PMCID: PMC6513812. 30456659 PMC6513812

[6] Huang Y, Wang W, Ren Q. Two host microRNAs influence WSSV replication via STAT gene regulation. Sci Rep. 2016;6:23643-. doi: 10.1038/srep23643 . 27029712 PMC4814834

[7] Kitab B, Alj HS, Ezzikouri S, Benjelloun S. MicroRNAs as important players in host-hepatitis B virus interactions. J Clin Transl Hepatol. 2015;3(2):149–61. Epub 2015/06/15. doi: 10.14218/JCTH.2015.00002 . 26357642 PMC4548348

[8] Dubey SK, Shrinet J, Sunil S. *Aedes aegypti* microRNA, miR-2944b-5p interacts with 3’UTR of chikungunya virus and cellular target vps-13 to regulate viral replication. PLoS Negl Trop Dis. 2019;13(6):e0007429. Epub 2019/06/05. doi: 10.1371/journal.pntd.0007429 ; PMCID: PMC6576790. 31166953 PMC6576790

[9] Sedano CD, Sarnow P. Hepatitis C virus subverts liver-specific miR-122 to protect the viral genome from exoribonuclease Xrn2. Cell Host Microbe. 2014;16(2):257–64. Epub 2014/08/15. doi: 10.1016/j.chom.2014.07.006 ; PubMed Central PMCID: PMC4227615. 25121753 PMC4227615

[10] Bruscella P, Bottini S, Baudesson C, Pawlotsky JM, Feray C, Trabucchi M. Viruses and miRNAs: More friends than foes. Front Microbiol. 2017;8:824. Epub 2017/05/31. doi: 10.3389/fmicb.2017.00824 ; PubMed Central PMCID: PMC5430039. 28555130 PMC5430039

[11] Cheng G, Liu Y, Wang P, Xiao X. Mosquito defense strategies against viral infection. Trends Parasitol. 2016;32(3):177–86. Epub 2015/12/03. doi: 10.1016/j.pt.2015.09.009 ; PubMed Central PMCID: PMC4767563. 26626596 PMC4767563

[12] Colpitts TM, Cox J, Vanlandingham DL, Feitosa FM, Cheng G, Kurscheid S, et al. Alterations in the *Aedes aegypti* transcriptome during infection with West Nile, dengue and yellow fever viruses. PLoS Pathog. 2011;7(9):e1002189–e. Epub 2011/09/01. doi: 10.1371/journal.ppat.1002189 . 21909258 PMC3164632

[13] Zhao W, Yang P, Kang L, Cui F. Different pathogenicities of Rice stripe virus from the insect vector and from viruliferous plants. New Phytol. 2016;210(1):196–207. Epub 2015/11/21. doi: 10.1111/nph.13747 ; PubMed Central PMCID: PMC5063192. 26585422 PMC5063192

[14] Yang M, Xu Z, Zhao W, Liu Q, Li Q, Lu L, et al. Rice stripe virus-derived siRNAs play different regulatory roles in rice and in the insect vector *Laodelphax striatellus*. BMC Plant Biol. 2018;18(1):219. Epub 2018/10/06. doi: 10.1186/s12870-018-1438-7 ; PubMed Central PMCID: PMC6172784. 30286719 PMC6172784

[15] Hussain M, Asgari S. MicroRNA-like viral small RNA from Dengue virus 2 autoregulates its replication in mosquito cells. Proc Natl Acad Sci U S A. 2014;111(7):2746–51. Epub 2014/02/20. doi: 10.1073/pnas.1320123111 ; PubMed Central PMCID: PMC3932895. 24550303 PMC3932895

[16] Hussain M, Taft RJ, Asgari S. An insect virus-encoded microRNA regulates viral replication. J Virol. 2008;82(18):9164–70. Epub 2008/07/11. doi: 10.1128/JVI.01109-08 ; PubMed Central PMCID: PMC2546896. 18614632 PMC2546896

[17] Toriyama S. Rice stripe virus: prototype of a new group of viruses that replicate in plants and insects. Microbiol Sci. 1986;3(11):347–51. Epub 1986/11/01. . 2856619

[18] Xiong R, Wu J, Zhou Y, Zhou X. Identification of a movement protein of the tenuivirus rice stripe virus. J Virol. 2008;82(24):12304–11. Epub 2008/09/27. doi: 10.1128/JVI.01696-08 ; PubMed Central PMCID: PMC2593352. 18818319 PMC2593352

[19] Chen Q, Wei T. Cell biology during infection of plant viruses in insect vectors and plant hosts. Mol Plant Microbe Interact. 2020;33(1):18–25. Epub 2019/11/16. doi: 10.1094/MPMI-07-19-0184-CR . 31729283

[20] Xiao Y, Li Q, Wang W, Fu Y, Cui F. Regulation of RNA interference pathways in the insect vector Laodelphax striatellus by viral proteins of rice stripe virus. Viruses. 2021;13(8). Epub 2021/08/29. doi: 10.3390/v13081591 ; PubMed Central PMCID: PMC8402809. 34452456 PMC8402809

[21] Zhao W, Yu J, Jiang F, Wang W, Kang L, Cui F. Coordination between terminal variation of the viral genome and insect microRNAs regulates rice stripe virus replication in insect vectors. PLoS Pathog. 2021;17(3):e1009424. Epub 2021/03/11. doi: 10.1371/journal.ppat.1009424 . 33690727 PMC7984632

[22] Zhu J, Jiang F, Wang X, Yang P, Bao Y, Zhao W, et al. Genome sequence of the small brown planthopper, *Laodelphax striatellus*. Gigascience. 2017;6(12):1–12. Epub 2017/11/15. doi: 10.1093/gigascience/gix109 ; PubMed Central PMCID: PMC5740986. 29136191 PMC5740986

[23] Wang H, Garzon R, Sun H, Ladner KJ, Singh R, Dahlman J, et al. NF-kappaB-YY1-miR-29 regulatory circuitry in skeletal myogenesis and rhabdomyosarcoma. Cancer cell. 2008;14(5):369–81. Epub 2008/11/04. doi: 10.1016/j.ccr.2008.10.006 ; PMCID: PMC3829205. 18977326 PMC3829205

[24] Zhao G, Li Q, Wang A, Jiao J. YY1 regulates melanoma tumorigenesis through a miR-9 ~ RYBP axis. J Exp Clin Cancer Res. 2015;34(1):66. Epub 2015/06/24. doi: 10.1186/s13046-015-0177-y ; PMCID: PMC4511530. 26104682 PMC4511530

[25] Zhao W, Zhu J, Lu H, Zhu J, Jiang F, Wang W, et al. The nucleocapsid protein of rice stripe virus in cell nuclei of vector insect regulates viral replication. Protein Cell. 2021. Epub 2021/03/07. doi: 10.1007/s13238-021-00822-1 33675514 PMC7936609

[26] Lee Y, Kim M, Han J, Yeom K-H, Lee S, Baek SH, et al. MicroRNA genes are transcribed by RNA polymerase II. EMBO J. 2004;23(20):4051–60. Epub 2004/09/16. doi: 10.1038/sj.emboj.7600385 ; PMCID: PMC524334. 15372072 PMC524334

[27] Kamola PJ, Nakano Y, Takahashi T, Wilson PA, Ui-Tei K. The siRNA non-seed region and its target sequences are auxiliary determinants of off-target effects. PLoS Comput Biol. 2015;11(12):e1004656. Epub 2015/12/15. doi: 10.1371/journal.pcbi.1004656 ; PubMed Central PMCID: PMC4676691. 26657993 PMC4676691

[28] Zhao W, Li Q, Cui F. Potential functional pathways of plant RNA virus-derived small RNAs in a vector insect. Methods. 2019. Epub 2019/10/28. doi: 10.1016/j.ymeth.2019.10.006 31654749

[29] Yin Q, Wang X, Roberts C, Flemington EK, Lasky JA. Methylation status and AP1 elements are involved in EBV-mediated miR-155 expression in EBV positive lymphoma cells. Virology. 2016;494:158–67. Epub 2019/04/26. doi: 10.1016/j.virol.2016.04.005 ; PMCID: PMC4884481. 27110708 PMC4884481

[30] Schnettler E, Sterken MG, Leung JY, Metz SW, Geertsema C, Goldbach RW, et al. Noncoding flavivirus RNA displays RNA interference suppressor activity in insect and Mammalian cells. J Virol. 2012;86(24):13486–500. Epub 2012/10/05. doi: 10.1128/JVI.01104-12 ; PubMed Central PMCID: PMC3503047. 23035235 PMC3503047

[31] Lakatos L, Csorba T, Pantaleo V, Chapman EJ, Carrington JC, Liu YP, et al. Small RNA binding is a common strategy to suppress RNA silencing by several viral suppressors. EMBO J. 2006;25(12):2768–80. Epub 2006/05/25. doi: 10.1038/sj.emboj.7601164 ; PMCID: PMC1500863. 16724105 PMC1500863

[32] Lee S, Song J, Kim S, Kim J, Hong Y, Kim Y, et al. Selective degradation of host MicroRNAs by an intergenic HCMV noncoding RNA accelerates virus production. Cell Host Microbe. 2013;13(6):678–90. Epub 2013/06/19. doi: 10.1016/j.chom.2013.05.007 . 23768492

[33] Hussain M, Torres S, Schnettler E, Funk A, Grundhoff A, Pijlman GP, et al. West Nile virus encodes a microRNA-like small RNA in the 3’ untranslated region which up-regulates GATA4 mRNA and facilitates virus replication in mosquito cells. Nucleic Acids Res. 2012;40(5):2210–23. Epub 2011/11/15. doi: 10.1093/nar/gkr848 ; PubMed Central PMCID: PMC3300009. 22080551 PMC3300009

[34] McGeary SE, Lin KS, Shi CY, Pham TM, Bisaria N, Kelley GM, et al. The biochemical basis of microRNA targeting efficacy. Science. 2019;366(6472):eaav1741. Epub 2019/12/05. doi: 10.1126/science.aav1741 . 31806698 PMC7051167

[35] Jackson AL, Bartz SR, Schelter J, Kobayashi SV, Burchard J, Mao M, et al. Expression profiling reveals off-target gene regulation by RNAi. Nat Biotechnol. 2003;21(6):635–7. Epub 2003/05/18. doi: 10.1038/nbt831 . 12754523

[36] Alemán LM, Doench J, Sharp PA. Comparison of siRNA-induced off-target RNA and protein effects. RNA. 2007;13(3):385–95. Epub 2007/01/19. doi: 10.1261/rna.352507 . 17237357 PMC1800510

[37] Doench JG, Petersen CP, Sharp PA. siRNAs can function as miRNAs. Genes Dev. 2003;17(4):438–42. Epub 2003/02/26. doi: 10.1101/gad.1064703 ; PubMed Central PMCID: PMC195999. 12600936 PMC195999

[38] Seto E, Shi Y, Shenk T. YY1 is an initiator sequence-binding protein that directs and activates transcription *in vitro*. Nature. 1996;93(24):13571–6. Epub 1996/11/26. doi: 10.1073/pnas.93.24.13571 PMCID: PMC19346. 1720509

[39] Xia Y, Wei K, Yang FM, Hu LQ, Pan CF, Pan XL, et al. miR-1260b, mediated by YY1, activates KIT signaling by targeting SOCS6 to regulate cell proliferation and apoptosis in NSCLC. Cell Death Dis. 2019;10(2):112. Epub 2019/02/08. doi: 10.1038/s41419-019-1390-y ; PMCID: PMC6368632. 30737371 PMC6368632

[40] Ye Z, Li G, Kim C, Hu B, Jadhav RR, Weyand CM, et al. Regulation of miR-181a expression in T cell aging. Nat Commun. 2018;9(1):3060. Epub 2018/08/03. doi: 10.1038/s41467-018-05552-3 ; PubMed Central PMCID: PMC6076328. 30076309 PMC6076328

[41] Tang W, Zhou W, Xiang L, Wu X, Zhang P, Wang J, et al. The p300/YY1/miR-500a-5p/HDAC2 signalling axis regulates cell proliferation in human colorectal cancer. Nat Commun. 2019;10(1):663. Epub 2019/02/08. doi: 10.1038/s41467-018-08225-3 ; PubMed Central PMCID: PMC6368584. 30737378 PMC6368584

[42] Hampsey M. Molecular genetics of the RNA polymerase II general transcriptional machinery. Microbiol Mol Biol Rev. 1998;62(2):465–503. Epub 1998/06/01. doi: 10.1128/MMBR.62.2.465-503.1998 PMCID: PMC98922. 9618449 PMC98922

[43] Haberle V, Stark A. Eukaryotic core promoters and the functional basis of transcription initiation. Nat Rev Mol Cell Biol. 2018;19(10):621–637. Epub 2018/10/01. doi: 10.1038/s41580-018-0028-8 ; PMCID: PMC6205604. 29946135 PMC6205604

[44] Spitz F, Furlong EEM. Transcription factors: from enhancer binding to developmental control. Nat Rev Genet. 2012;13(9):613–626. Epub 2012/08/07. doi: 10.1038/nrg3207 . 22868264

[45] Lu S, Rodriguez M, Liao W. YY1 represses rat serum amyloid A1 gene transcription and is antagonized by NF-kappa B during acute-phase response. Mol. Cell. Biol. 14(9):6253–6263. Epub 1994/09/01. doi: 10.1128/mcb.14.9.6253-6263.1994 PMCID: PMC359152. 8065357 PMC359152

[46] Gordon S, Akopyan G, Garban H, Bonavida B. Transcription factor YY1: structure, function, and therapeutic implications in cancer biology. Oncogene. 2006;25(8):1125–1142. Epub 2006/02/01. doi: 10.1038/sj.onc.1209080 . 16314846

[47] Thomas MJ, Seto E. Unlocking the mechanisms of transcription factor YY1: are chromatin modifying enzymes the key? Gene. 1999;236(2):197–208. Epub 1999/08/01. doi: 10.1016/s0378-1119(99)00261-9 . 10452940

[48] Shi Y, Lee JS, Galvin KM. Everything you have ever wanted to know about Yin Yang 1…… Biochimica et Biophysica Acta. 1997;1332(2):F49–66. Epub 1997/04/01. doi: 10.1016/s0304-419x(96)00044-3 9141463

[49] Bushmeyer S, Park K, Atchison ML. Characterization of functional domains within the multifunctional transcription factor, YY1. The Journal of Biological Chemistry. 1995;270(50):30213–30220. Epub 1995/12/01. doi: 10.1074/jbc.270.50.30213 . 8530432

[50] Friedländer MR, Chen W, Adamidi C, Maaskola J, Einspanier R, Knespel S, et al. Discovering microRNAs from deep sequencing data using miRDeep. Nat Biotechnol. 2008;26(4):407–15. Epub 2008/04/09. doi: 10.1038/nbt1394 . 18392026

[51] John B, Enright AJ, Aravin A, Tuschl T, Sander C, Marks DS. Human microRNA targets. PLoS Biol. 2004;2(11):e363. Epub 2004/10/27. doi: 10.1371/journal.pbio.0020363 ; PubMed Central PMCID: PMC521178. 15502875 PMC521178

[52] Rehmsmeier M, Steffen P, Hochsmann M, Giegerich R. Fast and effective prediction of microRNA/target duplexes. RNA. 2004;10(10):1507–17. Epub 2004/09/24. doi: 10.1261/rna.5248604 ; PubMed Central PMCID: PMC1370637. 15383676 PMC1370637

[53] Zhao W, Lu L, Yang P, Cui N, Kang L, Cui F. Organ-specific transcriptome response of the small brown planthopper toward rice stripe virus. Insect Biochem Mol Biol. 2016;70:60–72. Epub 2015/12/19. doi: 10.1016/j.ibmb.2015.11.009 . 26678499

[54] Zhao W, Xu Z, Zhang X, Yang M, Kang L, Liu R, et al. Genomic variations in the 3’-termini of Rice stripe virus in the rotation between vector insect and host plant. New Phytol. 2018;219(3):1085–96. Epub 2018/06/09. doi: 10.1111/nph.15246 ; PubMed Central PMCID: PMC6055815. 29882354 PMC6055815

