# Supplementary figures and images for: Interaction between endogenous microRNAs and virus-derived small RNAs controls viral replication in insect vectors
Source: PLoS Pathog. 2022 Jul 7;18(7):e1010709. doi: 10.1371/journal.ppat.1010709 (PMC9295959; doi:10.1371/journal.ppat.1010709)

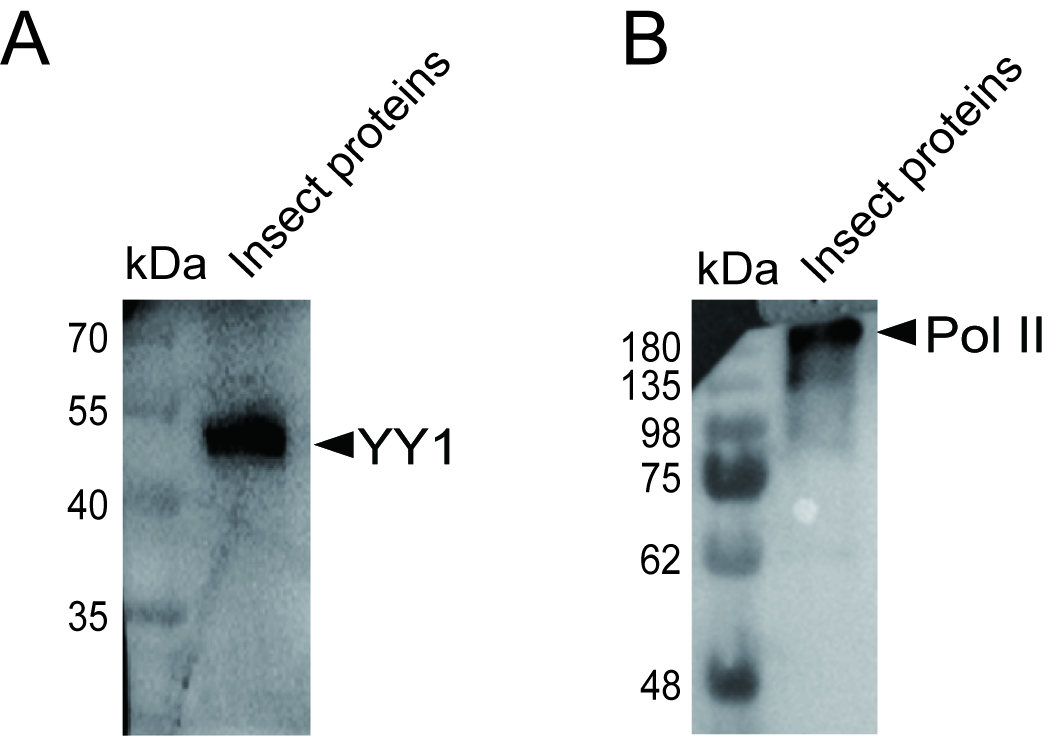

Supplement: S1 Fig — Verification of human anti-YY1 polyclonal antibody (A) and human anti-Pol II monoclonal antibody (B) in western blot analysis using the total proteins of small brown planthopper. (TIF) [file ppat.1010709.s001.tif]

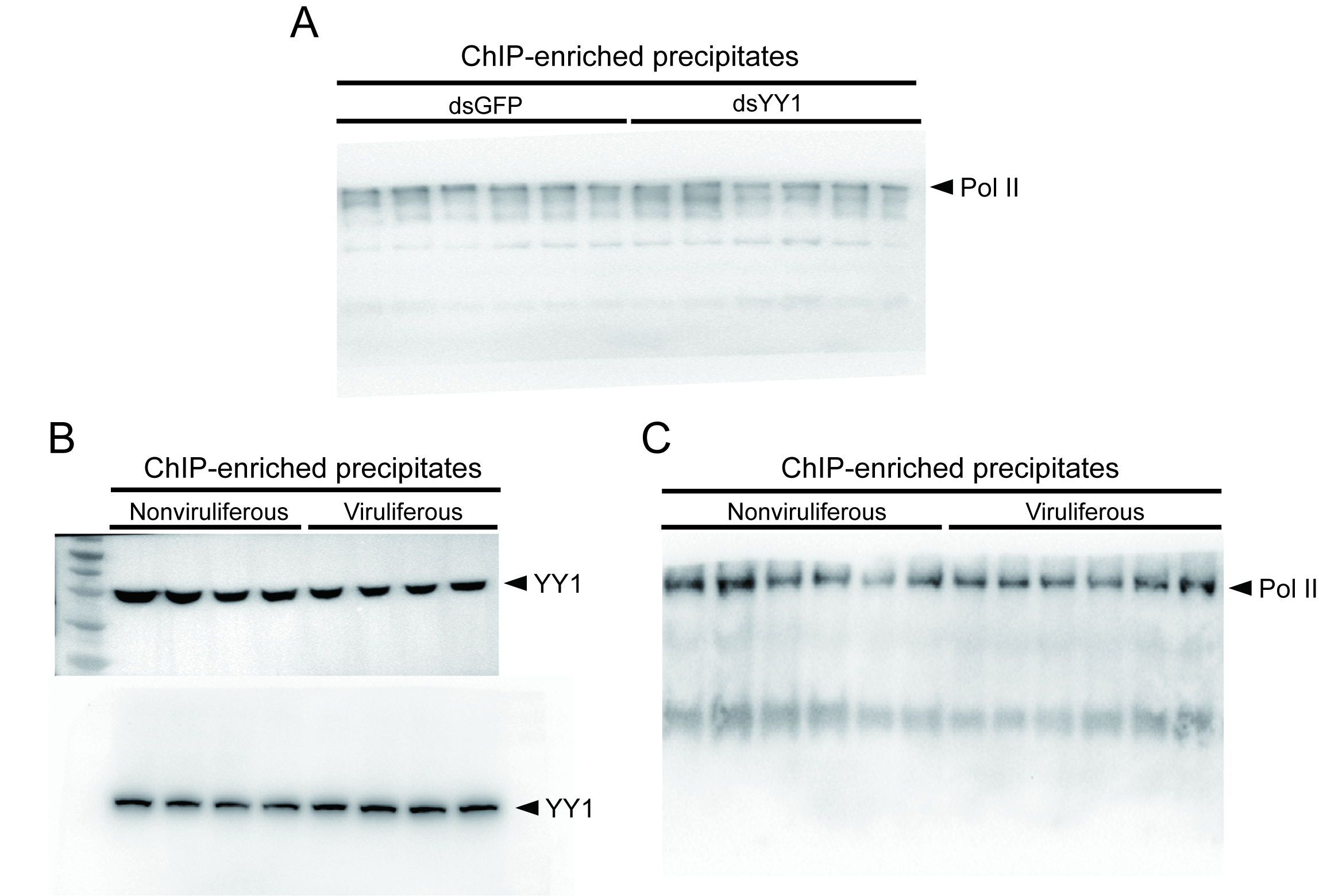

Supplement: S2 Fig — The complete images of western blots for ChIP analysis of Figs 2G (A), 3B (B) and 3C (C). (TIF) [file ppat.1010709.s002.tif]

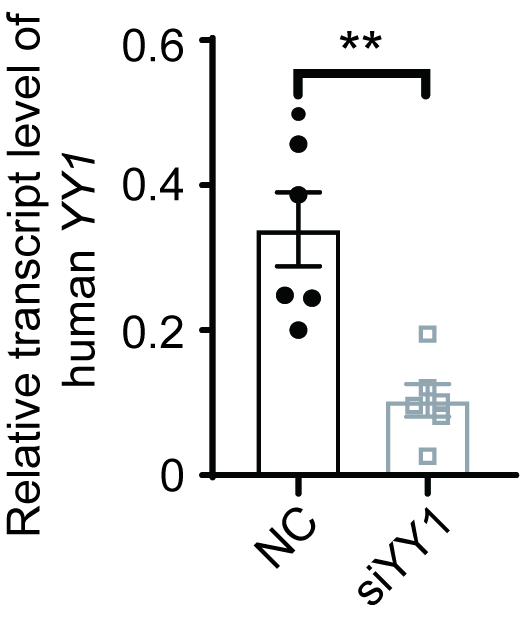

Supplement: S3 Fig — GFP siRNAs were used in the negative control group (NC). (TIF) [file ppat.1010709.s003.tif]

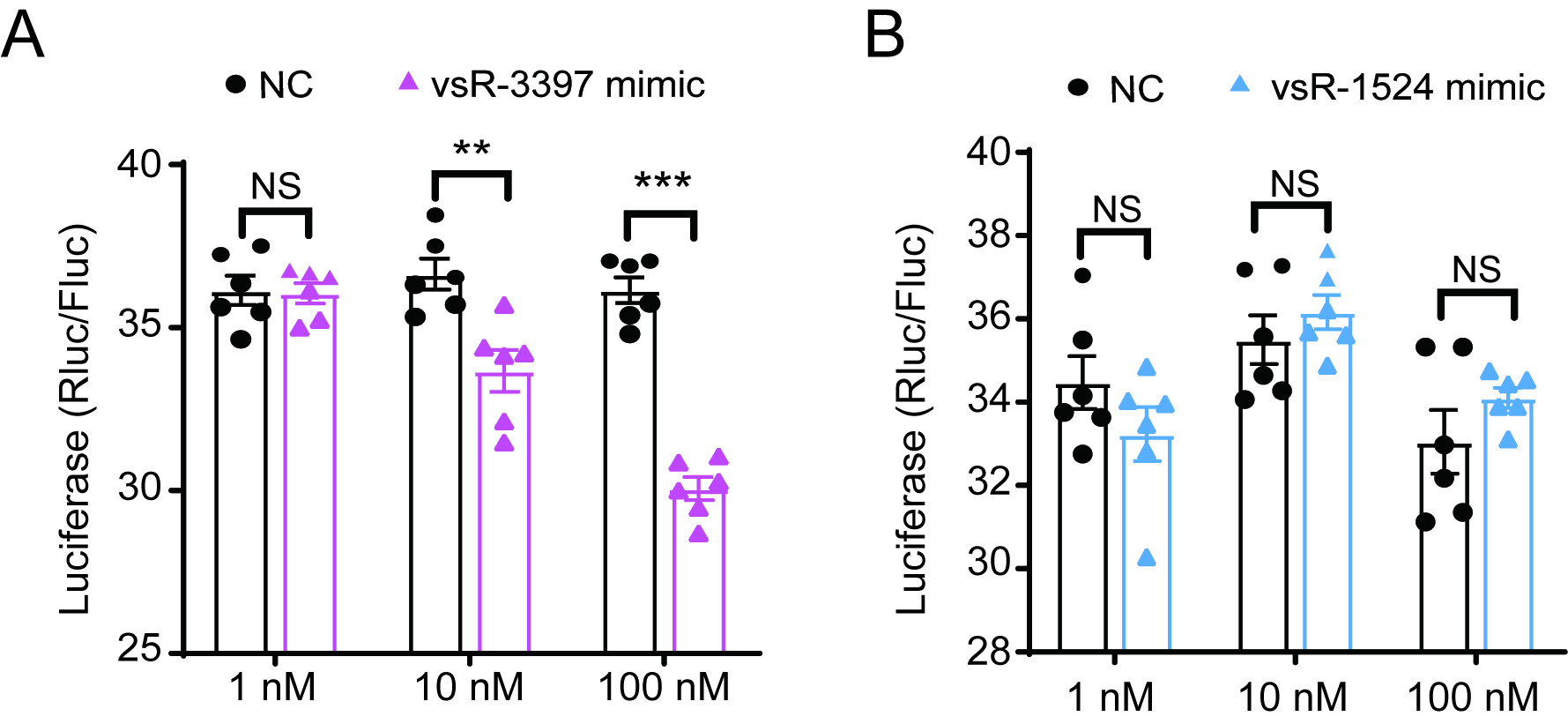

Supplement: S4 Fig — The activity of Renilla luciferase (Rluc) relative to that of firefly luciferase (Fluc) is presented (n = 6). NC, negative control. Values were compared by Student’s t test. NS, no significant difference. **, P<0.01. ***, P<0.001. (TIF) [file ppat.1010709.s004.tif]

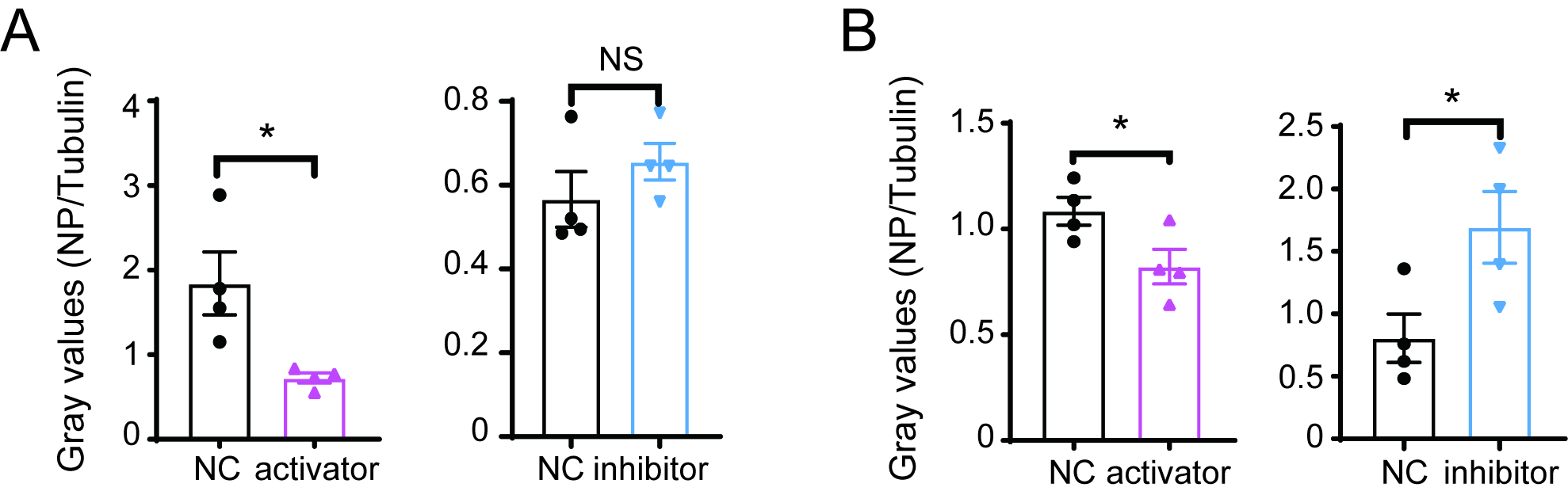

Supplement: S5 Fig — The relative optical densities of NP to that of Tubulin was calculated in the viruliferous planthoppers after injection with the vsR-3397 activator or inhibitor for 4 d (A), and in the nonviruliferous planthoppers after injection with the mixture of RSV and the activator or inhibitor of vsR-3397 for 6 d (B), corresponding to the western results of Fig 6A and 6B. (TIF) [file ppat.1010709.s005.tif]
