# Supplementary material for: Interaction between endogenous microRNAs and virus-derived small RNAs controls viral replication in insect vectors
Source: PLoS Pathog. 2022 Jul 7;18(7):e1010709. doi: 10.1371/journal.ppat.1010709 (PMC9295959; doi:10.1371/journal.ppat.1010709)
Supplement: S3 Table — (DOCX) [file ppat.1010709.s008.docx]

**S3 Table. Sequences of the primers and probes used in the study**

| **Sequence name** | **Sequence (5′ to 3′)** |
| --- | --- |
| primers for qPCR | |
| *Ls*-U6-F | TGGAACGATACAGAGAAGATTAGCA |
| *Ls*-U6-R | AACGCTTCACGATTTTGCGT |
| miR263-q-F | GGCGGGAATGGCACTGGAAGAAT |
| pre-miR263-q-F | CCCGTGGTCTTTCGGTGTCGTAAC |
| pri-miR263-q-F | GCTAATCTTCACTCACCTGAACAG |
| pri-miR263-q-R | GCCTTCATATTACGTATTTGCATTG |
| TTLL4-q-F | GGCCTCAACGAGCCTTACTCAAG |
| TTLL4-q-R | CTGGTTGAGTTGCTGGCAGCC |
| EF2-q-F | GTCTCCACGGATGGGCTTT |
| EF2-q-R | ATCTTGAATTTCTCGGCATACATTT |
| vsR-324-F | GCAACTGTGCTCTGTGACTCTG |
| vsR-1524-F | GGAGGATGTGTTGGTCTCTAGCT |
| vsR-2582-F | GCGATGTGCTTATCAGTGCCTCTGTA |
| vsR-3397-F | GCATCGTCTGTGGGTTCTGTGGA |
| vsR-3919-F | GCGGCTCTTATCTGGATTCTGTCCT |
| vsR-4630-F | CGTTTTGGGCTTTGTGTGTGATGA |
| vsR-5825-F | GGTCCTGTTGTACTGACTCCTATC |
| vsR-8778-F | GGTTTTGGGCTTTGTGTGTGATG |
| vsR-4170-F | CGCTTCTACTGTCCTTTGTACGTC |
| vsR-7858-F | GCGGGACGTACAAAGGACAGTAGAA |
| Primers for RNAi | |
| YY1-dsRNA-F | TGAGGGCAGTAACCCAGAA |
| YY1-dsRNA-R | CACATCAGCCACTTCCACC |
| GFP-dsRNA-F | CACAAGTTCAGCGTGTCCG |
| GFP-dsRNA-R | GTTCACCTTGATGCCGTTC |
| YY1-T7-F | TAATACGACTCACTATAGGTGAGGGCAGTAACCCAGAA |
| YY1-T7-R | TAATACGACTCACTATAGGCACATCAGCCACTTCCACC |
| GFP-T7-F | TAATACGACTCACTATAGGCACAAGTTCAGCGTGTCCG |
| GFP-T7-R | TAATACGACTCACTATAGGGTTCACCTTGATGCCGTTC |
| Primers for ChIP-qPCR | |
| YY1-TFBS1-F | ATGCGACTTTCCGTTCCGCG |
| YY1-TFBS1-R | TAACTCTCTCACCGGGCACCAG |
| YY1-TFBS2-F | CCTAAGTTCCAAATTACCTCTAAAC |
| YY1-TFBS2-R | TATCTCACTCCGAATTGCTGGC |
| Pol II-TSS-F | CAATGCGACTTTCCGTTCCGCG |
| Pol II-TSS-R | TGCATAACTCTCTCACCGGGCAC |
| Primers for RT-PCR | |
| TSS1-F1 | GAGTGGCTGTGACGGTGAAC |
| TSS1-R1 | GAATGAAGCGACCACGTACC |
| TSS1-F2 | CTAGAAACTCTGGAACAATG |
| TSS1-R2 | TACCGAAAACTGAAAGACA |
| Primers for expression plasmid construction | |
| NP-F | ATGGGTACCAACAAGCC |
| NP-R | CTAGTCATCTGCACCTTC |
| NP-NotI-F | GCGGCCGCATGGGTACCAAC |
| NP-XbaI-R | TCTAGACTAGTCATCTGCACCTTC |
| YY1-F | ATGGCGTCCGCTGATTACATTAC |
| YY1-R | ATCGGCATAGACAATGAACTGTTG |
| YY1-NheI-F | CGGCTAGCATGGCGTCCGCTGATTAC |
| YY1-KpnI-R | GGTACCTCAATCGGCATAGACAATG |
| Promoter1-F | CGTAGTGTACTATGATGTGG |
| Promoter1-R | GGATACAACGGTTCACTG |
| Promoter2-F | ATGAAATGCTAAAGCAGACTCC |
| Promoter2-R | GCAGTCAGAGAGCCCAATC |
| Promoter1-KpnI-F | GGGGTACCCGTAGTGTACTATGATG |
| Promoter1-NheI-R | GCTAGCGGATACAACGGTTCACTG |
| Promoter2-KpnI-F | GGGGTACCATGAAATGCTAAAGCAG |
| Promoter2-NheI-R | GCTAGCGCAGTCAGAGAGCCCA |
| vsR-1524-tar-F | GAGTGGCTGTGACGGTGAAC |
| vsR-1524-tar-R | CACCGAGAGAAAGAGAGAATATG |
| vsR-1524-tar-XhoI-F | CCGCTCGAGGAGTGGCTGTGAC |
| vsR-1524-tar-NotI-R | GCGGCCGCCACCGAGAGAAAG |
| vsR-3397-tar-F | CGGAGTGAGATATGATAATTGG |
| vsR-3397-tar-R | GCCTGTAGTTATTTGGTTTTGC |
| vsR-3397-tar-XhoI-F | CCGCTCGAGCGGAGTGAGATATG |
| vsR-3397-tar-NotI-R | GCGGCCGCGCCTGTAGTTATTTG |
| pC-vsR-F | CACAACCATTAGGTAACGATTTAAATAC |
| pC-vsR-R | GTTTTTCTATTATTGATATAGCATTTTTG |
| Probes synthesized for Northern blotting | |
| vsR-1524 | DIG-AGC+TAG+AGA+CCA+ACA+CAT+CCT-DIG |
| vsR-3397 | DIG-TCC+ACA+GAA+CCC+ACA+GAC+GAT+G-DIG |
| U6 snRNA | DIG-ATC+TTC+TCT+GTA+TCG+TTC+CA-DIG |
| Sequences synthesized for RNAi in 293T cells | |
| siYY1-74-F | AGAUCGAGGUGGAGACCAUTT |
| siYY1-74-R | AUGGUCUCCACCUCGAUCUTT |
| siYY1-128-F | AGGAGGAGGAGGAGGACGATT |
| siYY1-128-R | UCGUCCUCCUCCUCCUCCUTT |

^a^ F, forward primers; R, reverse primers.

^b^ +, the sites where locked nucleic acid (LNA) is added.
